# Supplementary material for: Assessing phototoxic drug properties of hydrochlorothiazide using human skin biopsies
Source: Commun Biol. 2025 May 6;8:705. doi: 10.1038/s42003-025-08064-1 (PMC12056033; doi:10.1038/s42003-025-08064-1)
Supplement: Supplementary file 1 — Supplemental materials [file 42003_2025_8064_MOESM1_ESM.pdf]

Online Supplementary Materials

Assessing Phototoxic Drug Properties of Hydrochlorothiazide

Using Human Skin Biopsies

Mathias Hohl[1], Felix Götzinger[1,2,3], Simone Jäger[1], Lea Wagmann[4], Mert Tokcan[1], Thomas Tschernig[5], Jörg Reichrath[6], Jan M. Federspiel[7], Peter Boor[8], Markus R. Meyer[4], Felix Mahfoud[1,2,3,#], Michael Böhm[1,#]

1. Department of Internal Medicine III – Cardiology, Angiology and Intensive Care Medicine, Saarland University Hospital, Saarland University, Homburg, Germany
2. Department of Cardiology, University Heart Center Basel, University Hospital Basel, Basel, Switzerland
3. Department of Biomedicine, University Hospital Basel and University of Basel, Basel, Switzerland
4. Department of Clinical and Experimental Toxicology & Pharmacology, Saarland University, Homburg, Germany
5. Institute of Anatomy and Cellbiology, Saarland University Hospital, Saarland University, Homburg, Germany
6. Department of Adult and Pediatric Dermatology, Venerology and Allergology Saarland University Hospital, Saarland University, Homburg, Germany
7. Institute of Legal Medicine, Saarland University, Faculty of Medicine, Homburg, Germany
8. Institute of Pathology, University Clinic, Aachen, Germany

these author share senior authorship

Address of correspondence:

Mathias Hohl PhD, Department of Internal Medicine III, Saarland University,

Kirrbergerstraße 100, 66421 Homburg

mathias.hohl@uks.eu

Supplementary material

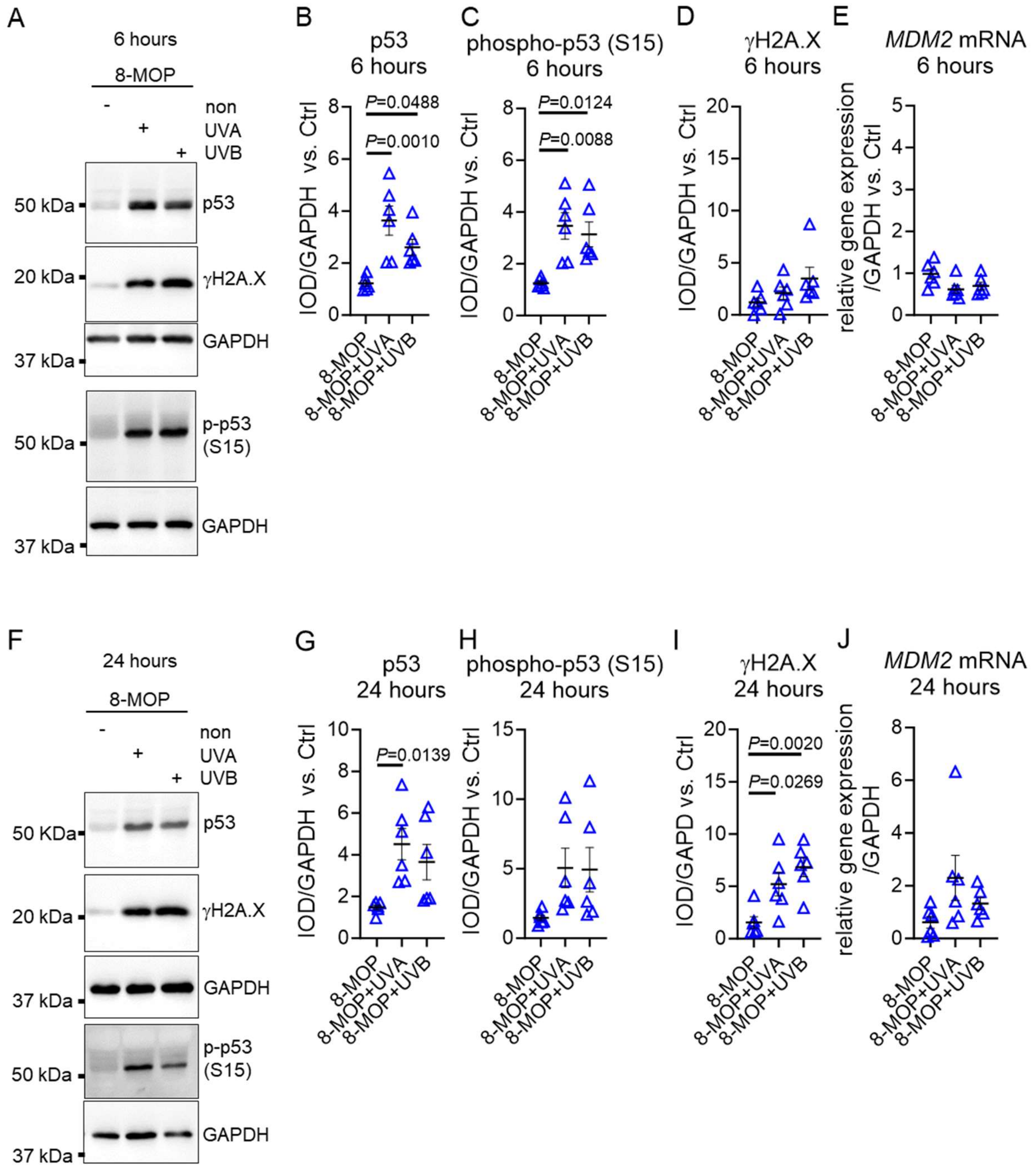

Supplementary Figure 1: Effect of UV-irradiation on γH2A.X, p53 protein, p53 phosphorylation and expression of *MDM2* mRNA in 8-MOP treated skin biopsies
(**A**) Representative Western blots demonstrating protein level of tumor suppressor protein p53, phosphorylation of histone H2A.X (γH2A.X) and phosphorylation of p53 (Serin15) in biopsies treated with 8-MOP (positive control) 6 hours after irradiation with 300 mJ/cm^2 UVA or UVB. Unirradiated biopsies served as group-specific control (non). Protein expression of Glyceraldehyde 3-Phosphate Dehydrogenase (GAPDH) served as loading control. Quantification of (**B**) p53 protein (**C**) phospho-p53, and (**D**) γH2A.X. (**E**) Gene expression of p53-regulator *MDM2* normalized against *GAPDH* 6 hours after irradiation. (**F**) Representative Western blots of p53, γH2A.X and phosphor-p53 (Serin15) in biopsies treated with 8-MOP (positive control) 24 hours after irradiation with 300 mJ/cm^2 UVA or UVB. Unirradiated

biopsies served as group-specific control (non). Protein expression of GAPDH served as loading control. Quantification of (**G**) p53 protein (**H**) phospho-p53, and (**I**) γH2A.X. (**J**) Gene expression of *MDM2* normalized against *GAPDH* 24 hours after irradiation. For (**B, C, D, E, G, H, I, J**) *n*=6 biopsies per group. Data are shown as mean±SEM with individual points. For comparison of three groups, P-value was determined using Kruskal-Wallis with Dunn´s multiple comparisons test for 8-MOP group in (**C, D, E**) and One-way ANOVA with Tukey multiple comparisons test for (**B, G, H, I, J**). IOD: Integrated optical density. Numerical source data are provided within the Supplementary Data 1 file.

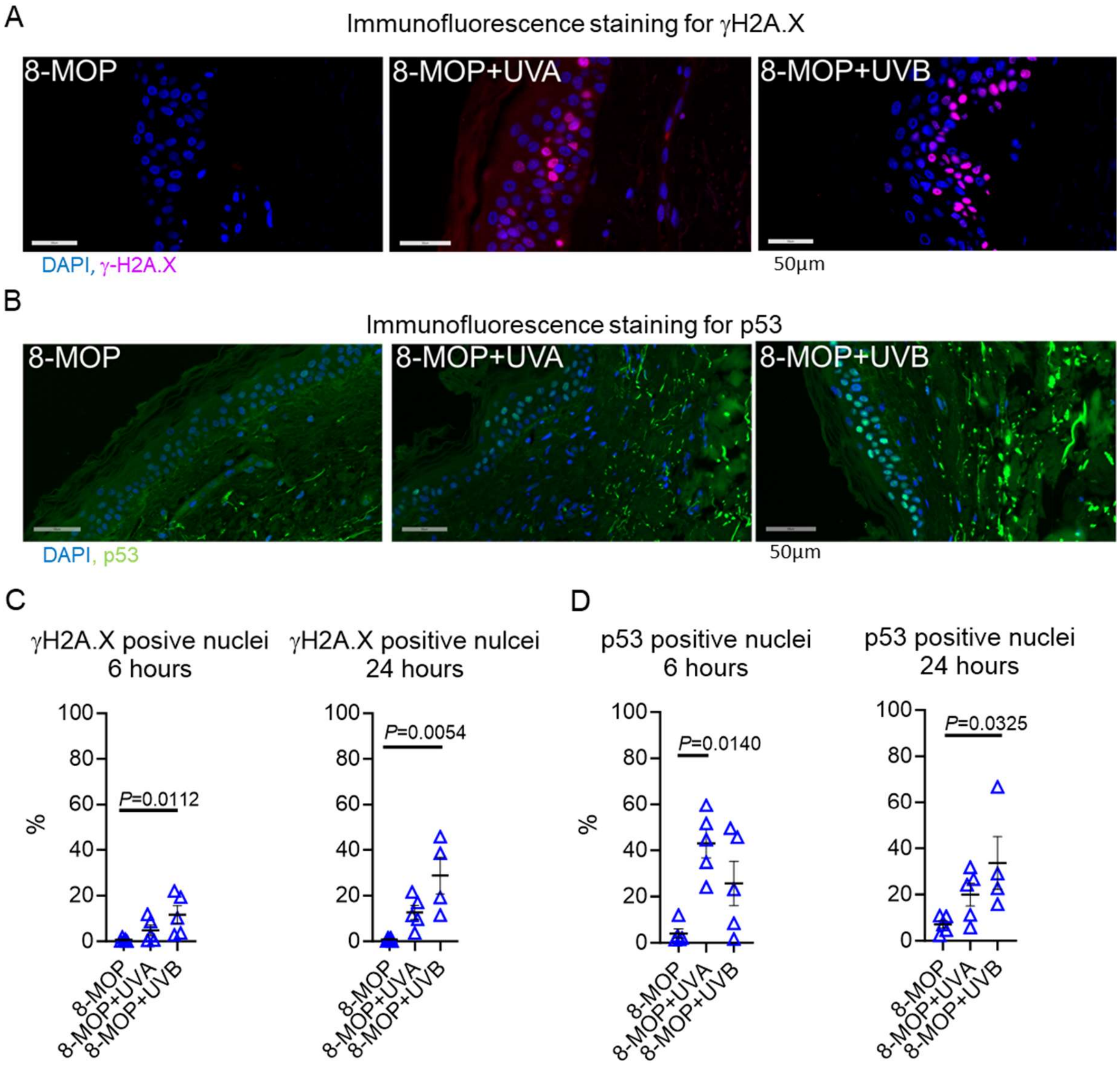

<u>Supplementary Figure 2:</u> Effect of UV-irradiation on nuclear expression of γH2A.X and p53 in 8-MOP-treated biopsies after UV irradiation

Representative immunostaining for γH2A.X and p53 of human skin biopsies 6 hours after UVA (**A**) or UVB (**B**) irradiation. (**C**) Quantification of γH2A.X positive stained nuclei 6 hours and 24 hours after UVA and UVB irradiation in the epidermis of 8-MOP treated skin biopsies. (**D**) Quantification of γH2A.X positive stained nuclei 6 hours and 24 hours after UVA and UVB irradiation in the epidermis of 8-MOP treated skin biopsies. For (**C, D**) *n*=5 biopsies per group except for 8-MOP+UVB 24 hours (**C, D**) *n*=4 biopsies. Data are shown as mean±SEM with individual data points. P-value was determined using Kruskal-Wallis with Dunn´s multiple comparisons test for comparison of three groups (**C, D**). Numerical source data are provided within the Supplementary Data 1 file.

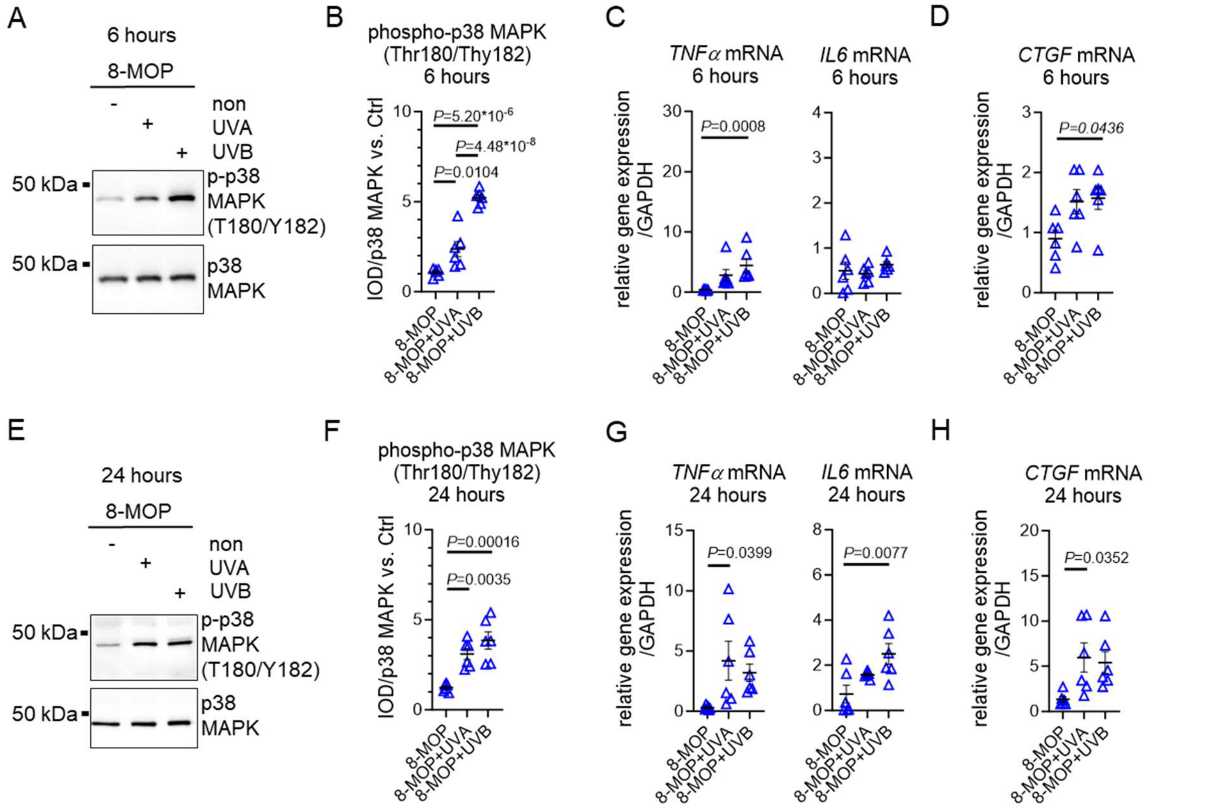

<u>Supplementary Figure 3:</u> Effect of UV-irradiation on p38 MAPK activation and pro-inflammatory response in 8-MOP-treated skin biopsies

(**A**) Representative Western blots demonstrating phosphorylation of p38 MAPK (T180/Y182) and total p38 MAPK protein in biopsies treated with 8-MOP (positive control) 6 hours after irradiation with 300 mJ/cm^2 UVA or UVB. Unirradiated biopsies served as group-specific control (non). (**B**) Quantification of phospho-p38 MAPK in relation to total p38 MAPK protein. (**C**) Gene expression of pro-inflammatory marker Tumor Necrosis Factor alpha (*TNFα*) and Interleukin 6 (*IL6*) and (**D**) Connective Tissue Growth Factor (*CTGF*) normalized against Glyceraldehyde 3-Phosphate Dehydrogenase (*GAPDH*) 6 hours after irradiation. (**E**) Representative Western blots demonstrating phosphorylation of p38 MAPK (T180/Y182) and total p38 MAPK protein in 8-MOP 24 hours after irradiation with 300 mJ/cm^2 UVA or UVB. Unirradiated biopsies served as group-specific control. (**F**) Quantification of phospho-p38 MAPK in relation to total p38 MAPK protein. (**G**) Gene expression of pro-inflammatory marker Tumor Necrosis Factor alpha (*TNFα*) and Interleukin 6 (*IL6*) and (**H**) Connective Tissue Growth Factor (*CTGF*) normalized against *GAPDH* 24 hours after irradiation. For **B, C, D, F, G, H** *n*=6 biopsies per group. Data are shown as mean±SEM with individual data points. For comparison of three groups, P-value was determined using Kruskal-Wallis with Dunn´s multiple comparisons test for 8-MOP group in (**C**) and One-way ANOVA with Tukey multiple comparisons test for (**B, D, F, G, H**). IOD: Integrated optical density. Numerical source data are provided within the Supplementary Data 1 file.

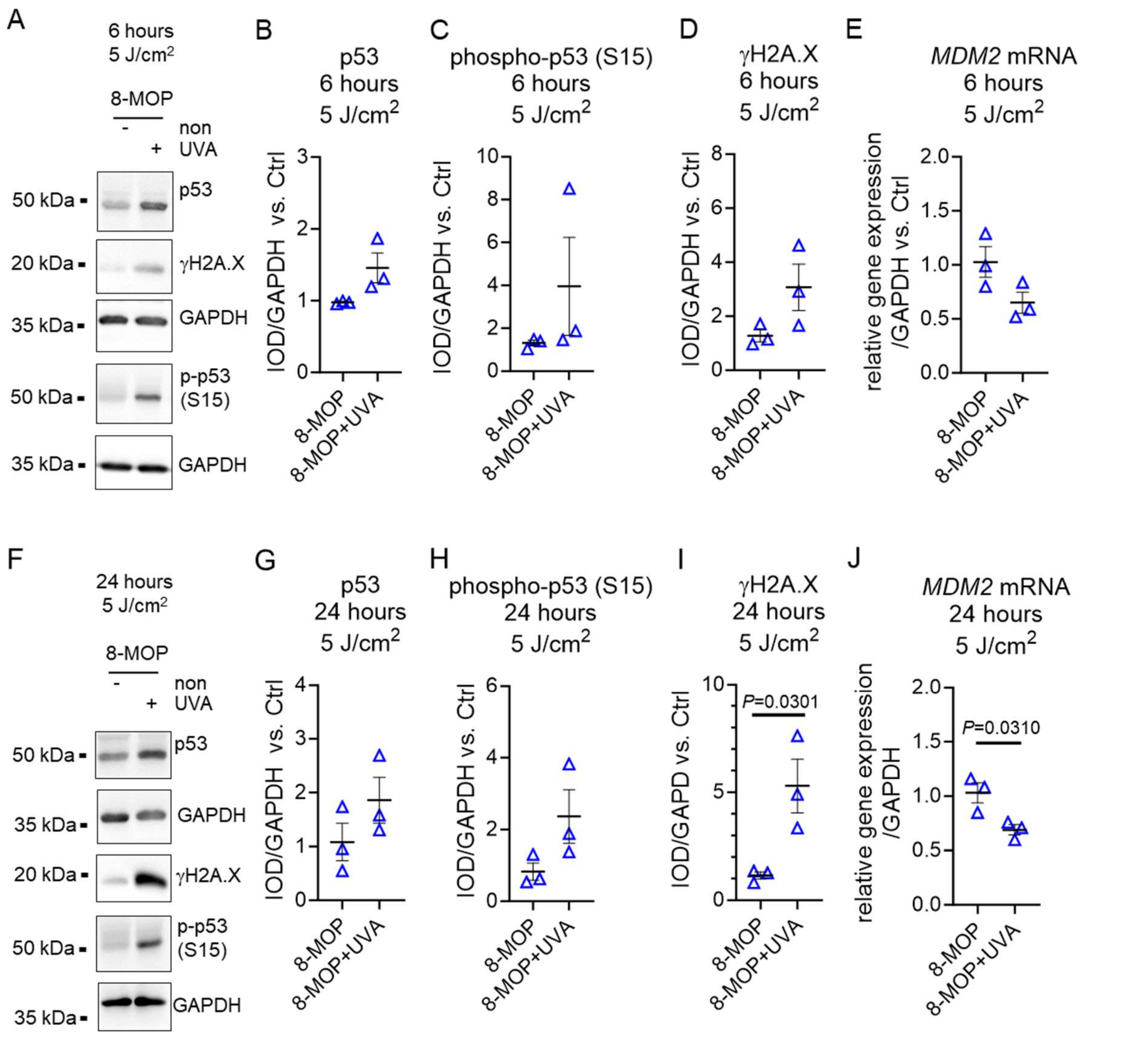

<u>Supplementary Figure 4:</u> Effect of irradiation with 5 J/cm^2 UVA on p53 protein, p53 phosphorylation, γH2A.X, and expression of *MDM2* mRNA in 8-MOP treated skin biopsies

(**A**) Representative Western blots demonstrating protein level of tumor suppressor protein p53, phosphorylation of histone H2A.X (γH2A.X) and phosphorylation of p53 (Serin15) in biopsies treated with 8-MOP (positive control) 6 hours after irradiation with 5 J/cm^2 UVA. Unirradiated biopsies served as group-specific control (non). Protein expression of Glyceraldehyde 3-Phosphate Dehydrogenase (GAPDH) served as loading control. Quantification of (**B**) p53 protein (**C**) phospho-p53, and (**D**) γH2A.X. (**E**) Gene expression of p53-regulator *MDM2* normalized against *GAPDH* 6 hours after irradiation. (**F**) Representative Western blots of p53, γH2A.X and phosphor-p53 (Serin15) in biopsies treated with 8-MOP (positive control) 24 hours after irradiation with 5 J/cm^2 UVA. Unirradiated biopsies served as group-specific control (non). Protein expression of GAPDH served as loading control. Quantification of (**G**) p53 protein (**H**) phospho-p53, and (**I**) γH2A.X. (**J**) Gene expression of *MDM2* normalized against *GAPDH* 24 hours after irradiation. For (**B, C, D, E, G, H, I, J**) n=3 biopsies per group. Data are shown as mean±SEM with individual points. P-value was determined using a Mann-Whitney test for comparison of groups for (**B, C, D, E, G, H, I, J**). Integrated optical density. Numerical source data are provided within the Supplementary Data 1 file.

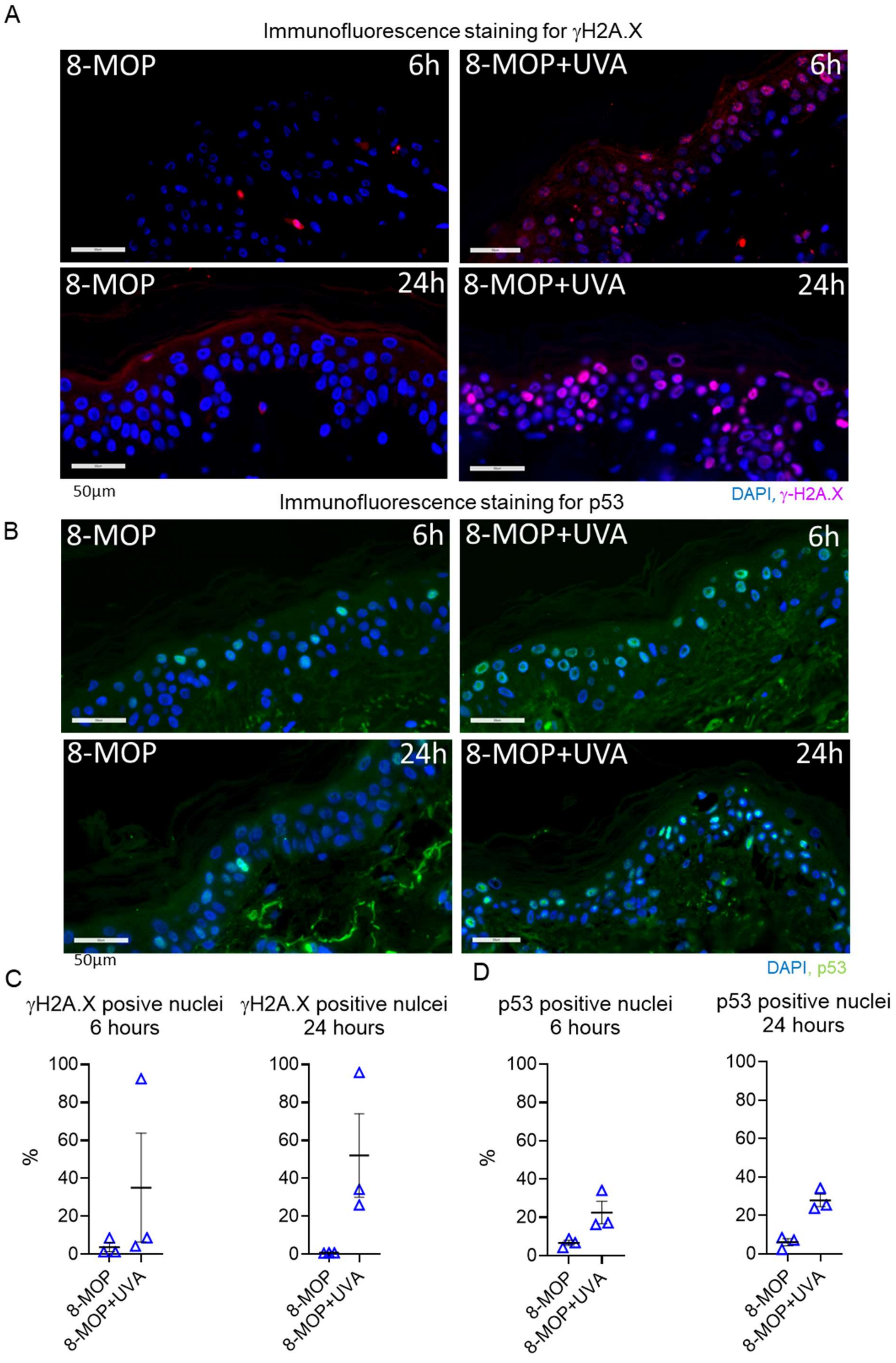
A
Immunofluorescence staining for γH2A.X
8-MOP
6h
8-MOP+UVA
6h
8-MOP
24h
8-MOP+UVA
24h
50µm
DAPI, γ-H2A.X
Immunofluorescence staining for p53
B
8-MOP
6h
8-MOP+UVA
6h
8-MOP
24h
8-MOP+UVA
24h
50µm
DAPI, p53
C
γH2A.X posive nuclei
6 hours
γH2A.X positive nulcei
24 hours
D
p53 positive nuclei
6 hours
p53 positive nuclei
24 hours
%
100
80
60
40
20
0
8-MOP
8-MOP+UVA

Supplementary Figure 5: Effect of irradiation with 5 J/cm^2 on nuclear expression of γH2A.X and p53 in 8-MOP-treated biopsies

(**A**) Representative immunostaining for γH2A.X of human skin biopsies six and 24 hours after irradiation with 5 J/cm^2 UVA. (**B**) Representative immunostaining for tumor suppressor protein p53 of human skin biopsies six and 24 hours after irradiation with 5 J/cm^2 UVA (**C**) Quantification of γH2A.X positive stained nuclei 6 hours and 24 hours after UVA irradiation in the epidermis of 8-MOP treated skin biopsies. (**D**) Quantification of p53 positive stained nuclei 6 hours and 24 hours after UVA irradiation in the epidermis of 8-MOP treated skin biopsies. For (**C, D**) n=3 biopsies per group. Data are shown as mean±SEM with individual data points. Mann-Whitney test was used for comparison of groups for (**C, D**). Numerical source data are provided within the Supplementary Data 1 file.

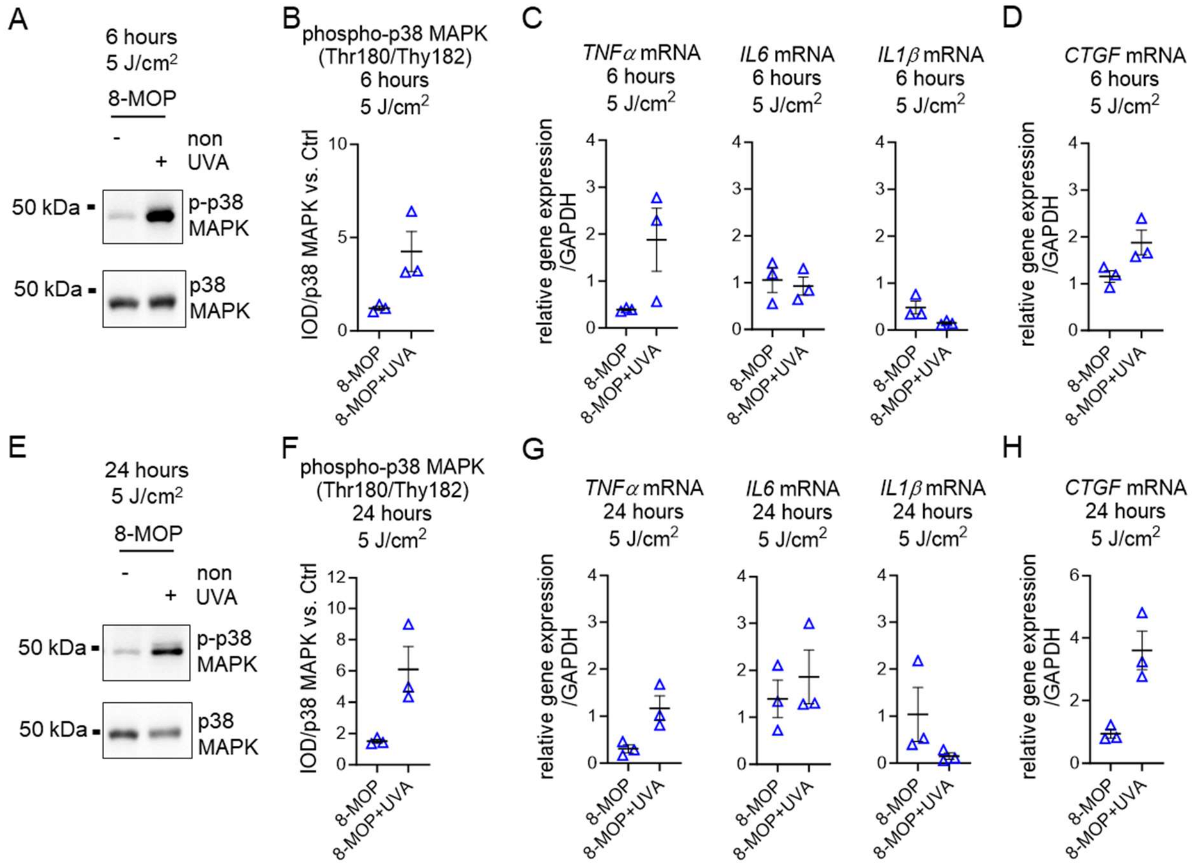

<u>Supplementary Figure 6:</u> Effect of irradiation with 5 J/cm^2 UVA on p38 MAPK activation and pro-inflammatory response in 8-MOP-treated skin biopsies.
(**A**) Representative Western blots demonstrating phosphorylation of p38 MAPK (T180/Y182) and total p38 MAPK protein in biopsies treated with 8-MOP (positive control) 6 hours after irradiation with 5 J/cm^2 UVA. Unirradiated biopsies served as group-specific control (non). (**B**) Quantification of phospho-p38 MAPK in relation to total p38 MAPK protein. (**C**) Gene expression of pro-inflammatory marker Tumor Necrosis Factor alpha (*TNFα*), Interleukin 6 (*IL6*), Interleukin 1β (*IL1β*) and (**D**) Connective Tissue Growth Factor (*CTGF*) normalized against Glyceraldehyde 3-Phosphate Dehydrogenase (*GAPDH*) 6 hours after irradiation. (**E**) Representative Western blots demonstrating phosphorylation of p38 MAPK (T180/Y182) and total p38 MAPK protein in 8-MOP 24 hours after irradiation with 5 J/cm^2 UVA. Unirradiated biopsies served as group-specific control. (**F**) Quantification of phospho-p38 MAPK in relation to total p38 MAPK protein. (**G**) Gene expression of pro-inflammatory marker Tumor Necrosis Factor alpha (*TNFα*), Interleukin 6 (*IL6*), Interleukin 1β (*IL1β*) and (**H**) Connective Tissue Growth Factor (*CTGF*) normalized against *GAPDH* 24 hours after irradiation. Data are shown as mean±SEM with individual data points. For (**B, C, D, F, G, H**) n=3 biopsies per group. For comparison of 2 groups a Mann-Whitney test was used for (**B, C, D, F, G, H**). Numerical source data are provided within the Supplementary Data 1 file.

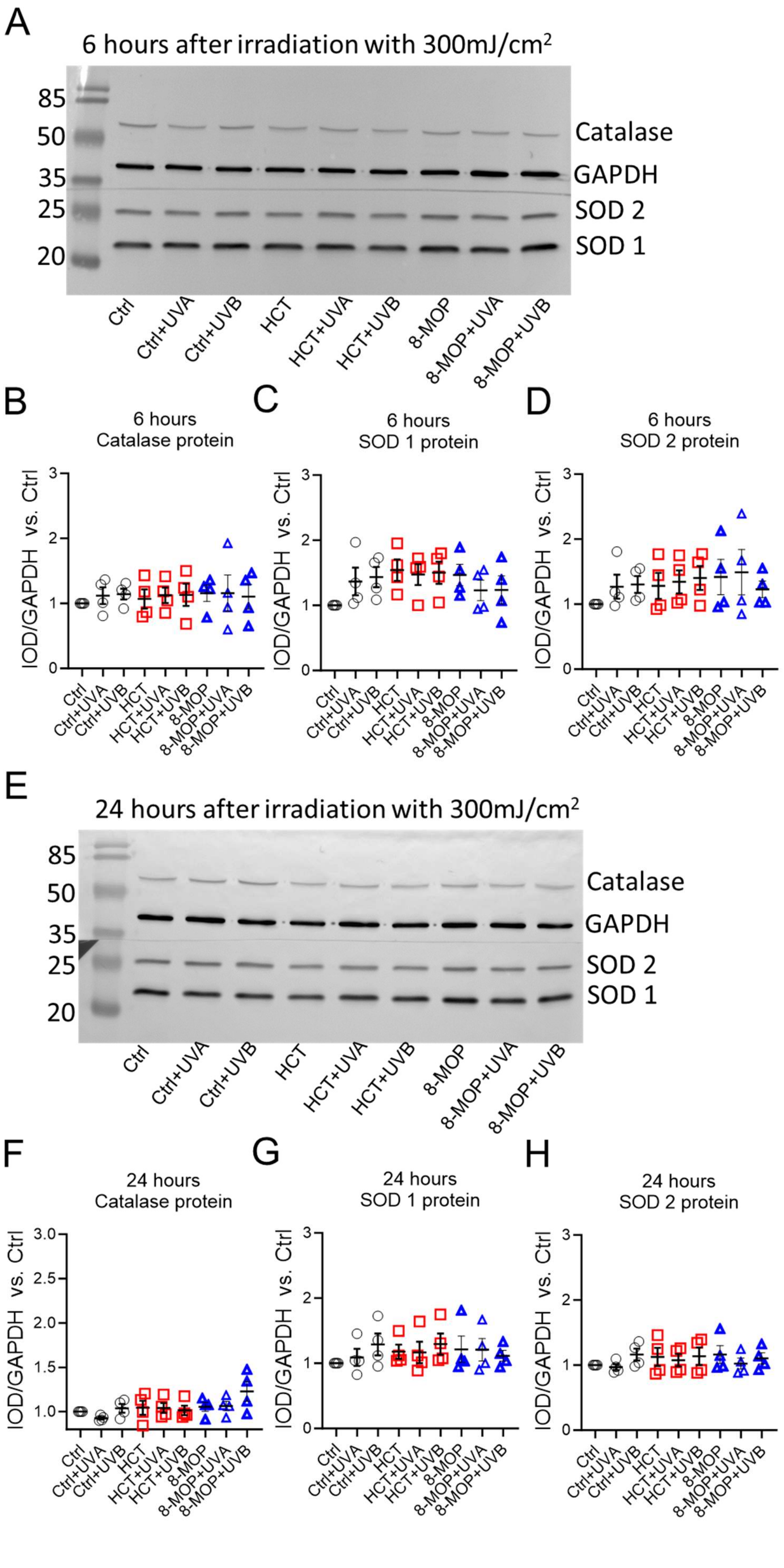

A
6 hours after irradiation with 300mJ/cm2
85
50
35
25
20
Catalase
GAPDH
SOD 2
SOD 1
Ctrl
Ctrl+UVA
Ctrl+UVB
HCT
HCT+UVA
HCT+UVB
8-MOP
8-MOP+UVA
8-MOP+UVB
B
6 hours
Catalase protein
C
6 hours
SOD 1 protein
D
6 hours
SOD 2 protein
IOD/GAPDH vs. Ctrl
E
24 hours after irradiation with 300mJ/cm2
F
24 hours
Catalase protein
G
24 hours
SOD 1 protein
H
24 hours
SOD 2 protein

Supplementary Figure 7: Effect of UV-irradiation on expression of anti-oxidative capacity proteins catalase, superoxide dismutase 1 (SOD 1) and SOD 2 after six and 24 hours. (**A**) Representative uncropped Western blots demonstrating protein expression of catalase, SOD 1 and SOD 2 in untreated (Ctrl), HCT-treated and 8-MOP-treated (positive control) biopsies 6 hours after irradiation with 300 mJ/cm^2 UVA or UVB. Unirradiated biopsies served as group-specific control. Quantification of (**B**) catalase, (**C**) SOD 1 and (**D**) SOD 2 normalized against Glyceraldehyde 3-Phosphate Dehydrogenase (GAPDH) 6 hours after irradiation. (**E**) Representative uncropped Western blots demonstrating protein expression of catalase, SOD 1 and SOD 2 in untreated (Ctrl), HCT-treated and 8-MOP-treated (positive control) biopsies 24 hours after irradiation with 300 mJ/cm^2 UVA or UVB. Unirradiated biopsies served as group-specific control. Quantification of (**F**) catalase, (**G**) SOD 1, and (**H**) SOD 2 normalized against Glyceraldehyde 3-Phosphate Dehydrogenase (GAPDH) 24 hours after irradiation. For **B, C, D, F, G, H** n=4 biopsies per group. Data are shown as mean±SEM with individual data points. For comparison of three groups, P-value was determined using Kruskal-Wallis with Dunn´s multiple comparisons test for all groups in (**B, C, D**, **F, G, H**) IOD: Integrated optical density. Numerical source data are provided within the Supplementary Data 1 file.

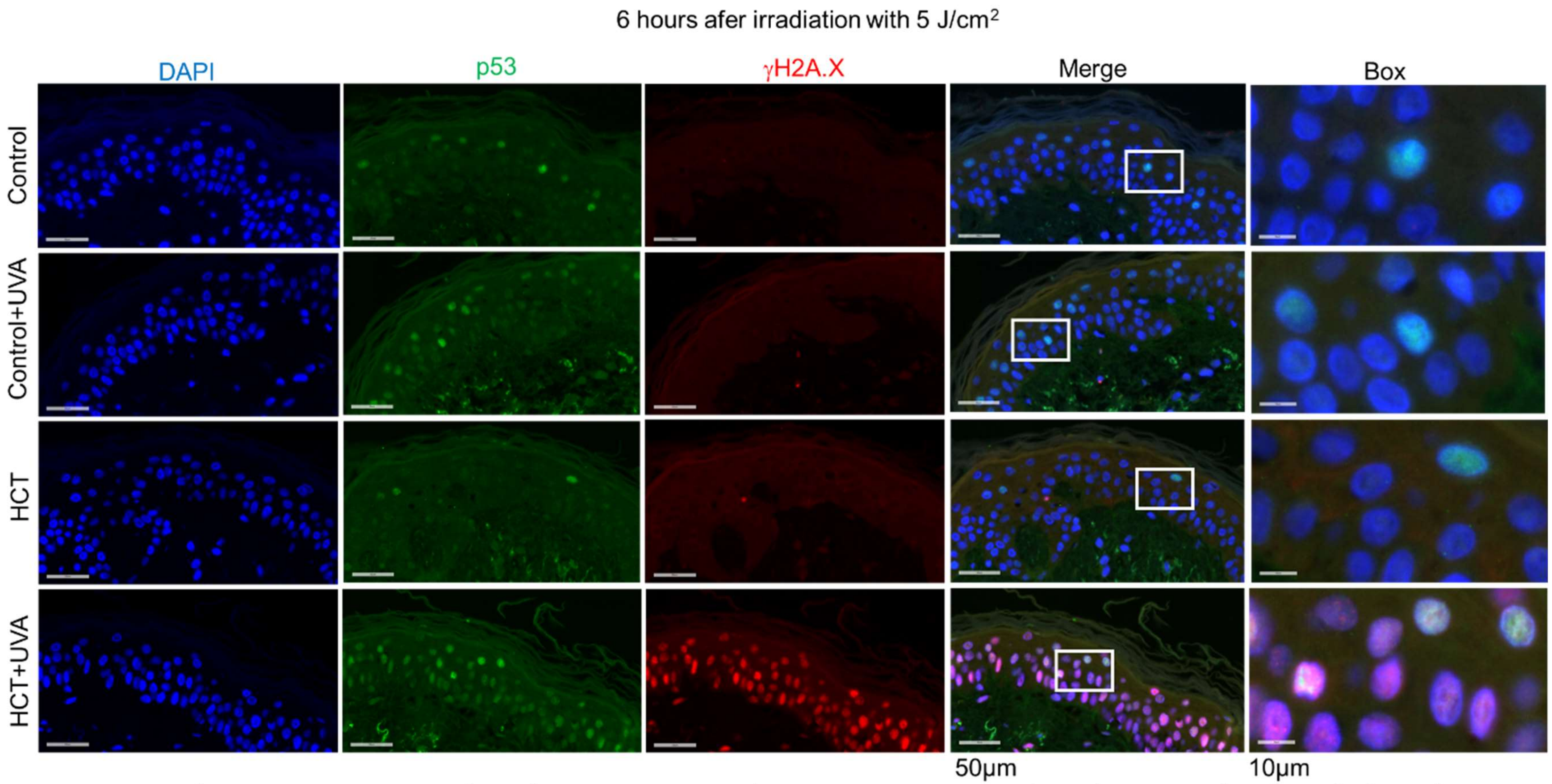

Supplementary Figure 8: Nuclear location of p53 and the DNA damage marker γH2A.X six hours after irradiation with 5J/cm^2 in Control (Ctrl) and HCT-treated skin biopsies.

Representative immunofluorescence images of DAPI (nuclei in blue), p53 (green), γH2A.X (red) and merged images of stainings (scale bar 50µm). White box shows scale bar 10µm

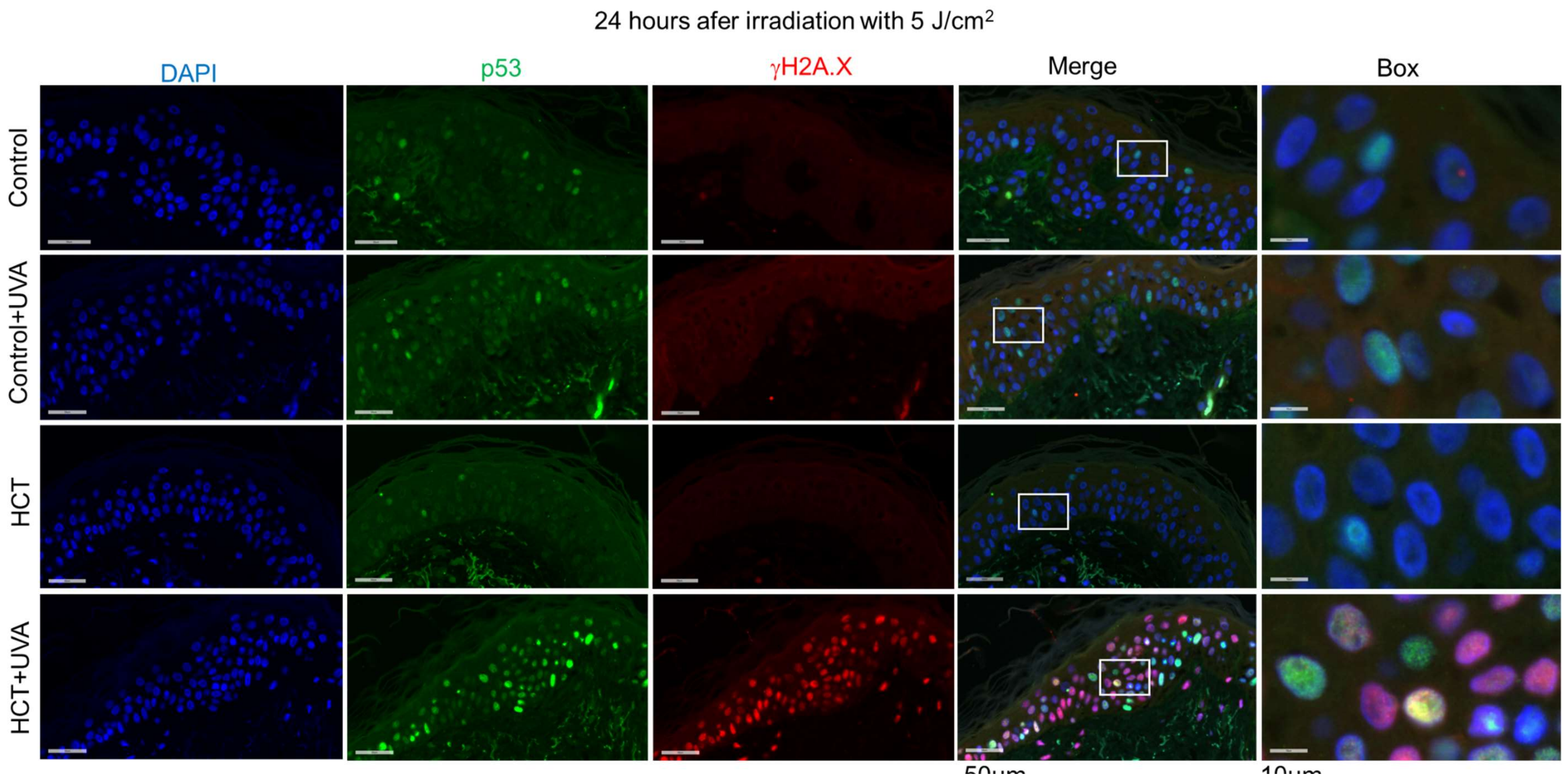

Supplementary Figure 9: Nuclear location of p53 and the DNA damage marker γH2A.X 24 hours after irradiation with 5J/cm^2 in Control (Ctrl) and HCT-treated skin biopsies.
Representative immunofluorescence images of DAPI (nuclei in blue), p53 (green), γH2A.X (red) and merged images of stainings (scale bar 50µm). White box shows scale bar 10µm

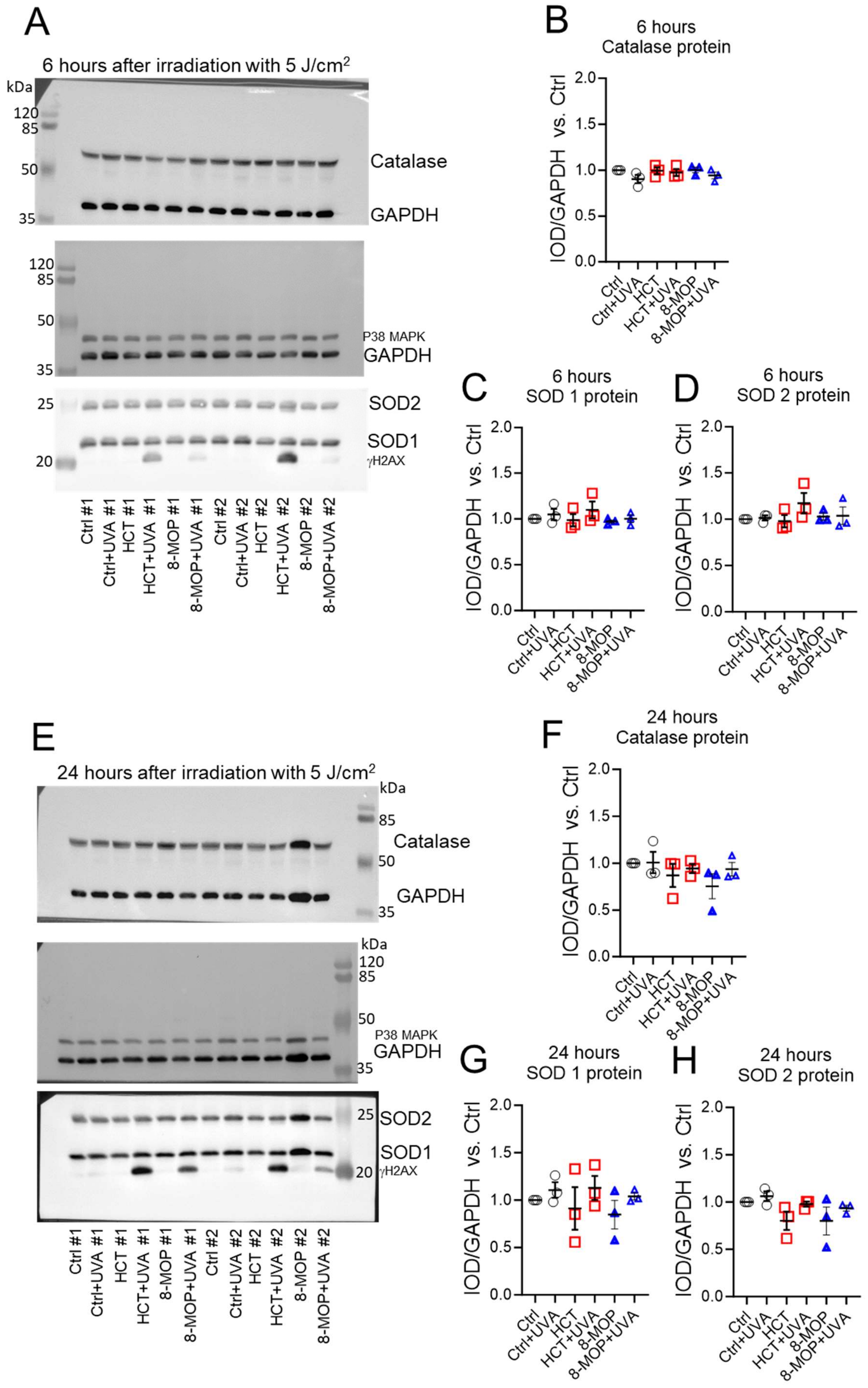

A
6 hours after irradiation with 5 J/cm2
kDa
120
85
50
35
Catalase
GAPDH
P38 MAPK
GAPDH
25
20
SOD2
SOD1
γH2AX
Ctrl #1
Ctrl+UVA #1
HCT #1
HCT+UVA #1
8-MOP #1
8-MOP+UVA #1
Ctrl #2
Ctrl+UVA #2
HCT #2
HCT+UVA #2
8-MOP #2
8-MOP+UVA #2
B
6 hours
Catalase protein
C
6 hours
SOD 1 protein
D
6 hours
SOD 2 protein
IOD/GAPDH vs. Ctrl
Ctrl
Ctrl+UVA
HCT
HCT+UVA
8-MOP
8-MOP+UVA
E
24 hours after irradiation with 5 J/cm2
F
24 hours
Catalase protein
G
24 hours
SOD 1 protein
H
24 hours
SOD 2 protein

<u>Supplementary Figure 10:</u> Effect of irradiation with high-dose 5 J/cm^2 UVA on expression of anti-oxidative capacity proteins catalase, superoxide dismutase 1 (SOD 1) and SOD 2 after six and 24 hours in untreated (Ctrl), HCT-treated and 8-MOP treated biopsies.
(**A**) Representative uncropped Western blots demonstrating protein expression of catalase, SOD 1 and SOD 2 in untreated (Ctrl), HCT-treated and 8-MOP-treated (positive control) biopsies 6 hours after irradiation with 5 J/cm^2 UVA. Unirradiated biopsies served as group-specific control. Quantification of (**B**) catalase, (**C**) SOD 1 and (**D**) SOD 2 normalized against Glyceraldehyde 3-Phosphate Dehydrogenase (GAPDH) 6 hours after irradiation. (**E**) Representative uncropped Western blots demonstrating protein expression of catalase, SOD 1 and SOD 2 in untreated (Ctrl), HCT-treated and 8-MOP-treated (positive control) biopsies 24 hours after irradiation with 5 J/cm^2 UVA. Unirradiated biopsies served as group-specific control. Quantification of (**F**) catalase, (**G**) SOD 1 and (**H**) SOD 2 normalized against Glyceraldehyde 3-Phosphate Dehydrogenase (GAPDH) 24 hours after irradiation. For **B, C, D, F, G, H** n=3 biopsies per group. Data are shown as mean±SEM with individual data points. IOD: Integrated optical density. For comparison of groups, P-value was determined using Kruskal-Wallis with Dunn´s multiple comparisons test for all groups in (**B, C, D, F, G, H**). Numerical source data are provided within the Supplementary Data 1 file.

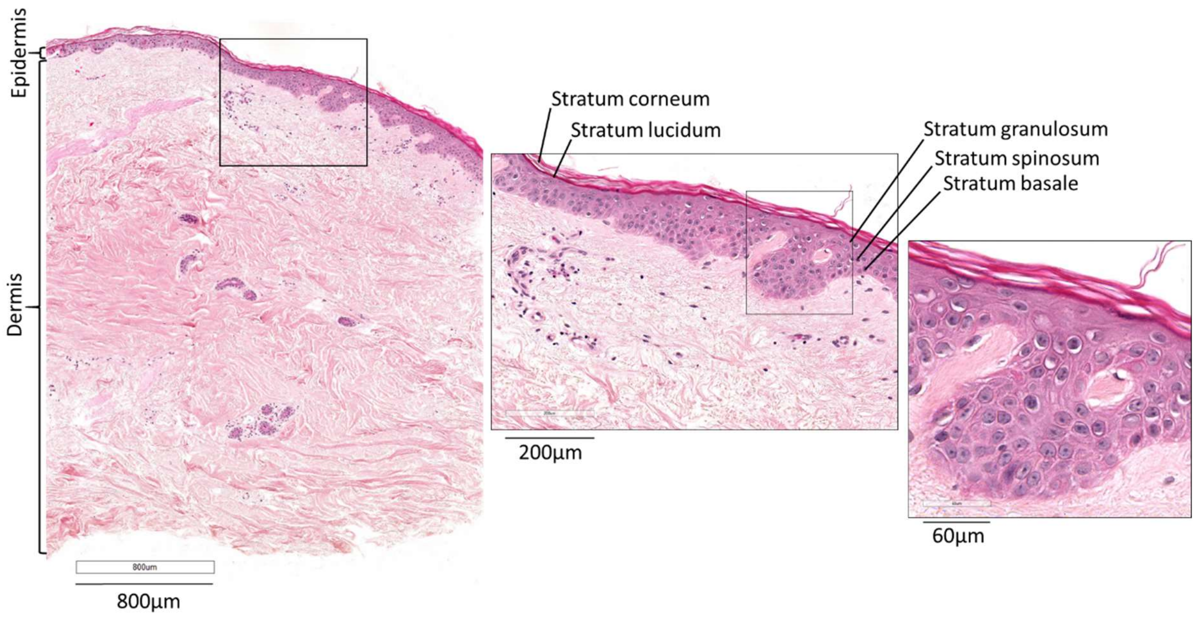

<u>Supplementary Figure 11:</u> Overview staining of human skin biopsy.
Representative image of hematoxylin and eosin stained biopsy demonstrating that sampled biopsies comprises the epidermis and dermis.

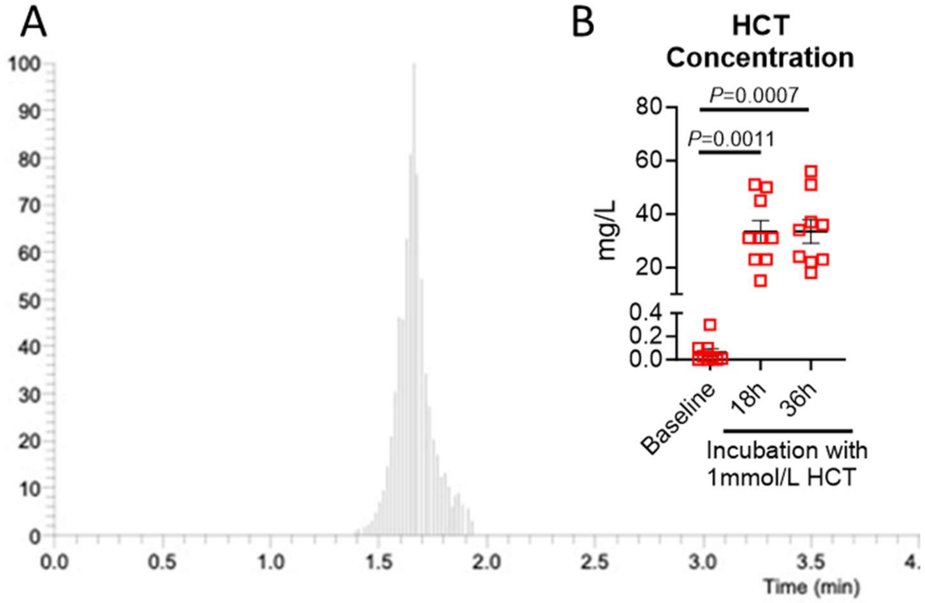

<u>Supplementary Figure 12</u>: Quantification of HCT in human skin biopsies using ultra-high-performance liquid chromatography and high-resolution tandem mass spectrometry (LC-HRMS/MS)

Toxicological analysis of human skin biopsies to confirm bioavailability of HCT at baseline (prior to incubation), 18 hours and 36 hours after incubation with 1mmol/HCT dissolved in DMSO. *n*=9 biopsies per groups. Data are shown as mean±SEM with individual data points. For comparison of three groups, P-value was determined using One-way ANOVA with Tukey multiple comparisons test. Numerical source data are provided within the Supplementary Data 1 file.

Supplementary Table 1: Altered mRNA expression of marker genes involved in apoptosis and tumorigenesis following UV-irradiation in 8-MOP treated skin biopsies

	8-MOP	8-MOP+UVA	8-MOP+UVB
Relative gene expression/GAPDH	6 hours after irradiation with 300 mJ/cm^2		
Vitamin D-Receptor (*VDR*)	1.13±0.15	0.42±0.04 *P*=0.0003vs. 8-MOP	0.68±0.06 *P*=0.0150 vs. 8-MOP
P2X7-Receptor (*P2RX7*)	1.09±0.2	0.33±0.03 *P*=0.0008 vs. 8-MOP	0.46±0.06 *P*=0.0040 vs. 8-MOP
Bcl-2	1.19±0.1	0.57±0.05 *P*=0.0086 vs. 8-MOP	0.96±0.08
Bak1	2.10±1.1	0.97±0.4	1.35±0.6
Bax	1.06±0.1	0.78±0.06	1.13±0.16
Relative gene expression/GAPDH	24 hours after irradiation with 300 mJ/cm^2		
Vitamin D-Receptor (*VDR*)	1.05±0.04	0.54±0.1 *P*=0.0006 vs. 8-MOP	0.43±0.04 *P*=0.00008 vs. 8-MOP
P2X7-Receptor (*P2RX7*)	0.76±0.09	0.42±0.04 *P*=0.0043 vs. 8-MOP	0.30±0.03 *P*=0.0002 vs. 8-MOP
Bcl-2	0.67±0.1	0.55±0.1	0.67±0.1
Bak1	1.15±0.2	0.80±0.2	0.55±0.1 *P*=0.0327 vs. 8-MOP
Bax	1.11±0.1	1.25±0.2	0.98±0.2

Data are shown as mean±SEM. For Vitamin D-Receptor (*VDR*) and P2X7-Receptor (*P2RX7*) *n*=6 biopsies per group, for *Bcl-2*, *Bak1*, and *Bax* *n*=5 biopsies per group. For comparison of three groups P-value was determined using Kruskal-Wallis with Dunn´s multiple comparisons test for *Bcl-2*, *Bak1* and *Bax*. One-way ANOVA with Tukey multiple comparisons test was used for P2X7 receptor at 6 hours and 24 hours and Vitamin D-Receptor at 6 and 24 hours. Numerical source data are provided within the Supplementary Data 1 file.

Supplementary Table 2: Effect of irradiation with high-dose 5 J/cm^2 on mRNA expression of marker genes involved in apoptosis and tumorigenesis in 8-MOP-treated skin biopsies

	8-MOP	8-MOP+UVA 5J/cm^2
Relative gene expression/GAPDH	6 hours after irradiation with 5 J/cm^2	
Vitamin D-Receptor (*VDR*)	1.27±0.01	0.78±0.07
P2X7-Receptor (*P2RX7*)	0.95±0.12	0.64±0.12
Bcl-2	0.89±0.03	0.80±0.06
Bak1	0.83±0.13	0.54±0.04
Bax	0.75±0.12	0.78±0.06
Relative gene expression/GAPDH	24 hours after irradiation with 5 J/cm^2	
Vitamin D-Receptor (*VDR*)	0.90±0.05	0.61±0.15
P2X7-Receptor (*P2RX7*)	0.76±0.15	0.30±0.04
Bcl-2	0.61±0.08	0.38±0.02
Bak1	0.91±0.08	0.36±0.12
Bax	1.00±0.04	0.69±0.12

Data are shown as mean±SEM. For Vitamin D-Receptor (*VDR*), P2X7-Receptor (*P2RX7*), *Bcl-2*, *Bak1*, and *Bax* n=3 biopsies per group. Data are shown as mean±SEM. For comparison of 2 groups a Mann-Whitney test was used. Numerical source data are provided within the Supplementary Data 1 file.

Supplementary Table 3: Effect of irradiation with high-dose 5 J/cm^2 on mRNA expression of marker genes involved in apoptosis and tumorigenesis

	Ctrl (n=3)	Ctrl +UVA (n=3)	HCT (n=3)	HCT +UVA (n=3)
Relative gene expression/GAPDH	6 hours after irradiation with 5 J/cm^2			
Vitamin D-Receptor (*VDR*)	1.00±0.003	1.09±0.15	1.01±0.02	0.48±0.03
P2X7-Receptor (*P2RX7*)	1.02±0.01	1.60±0.21	1.42±0.05	0.38±0.07 *P*=0.0389 vs. Ctrl+UVA
Bcl-2	1.01±0.01	1.13±0.13	1.09±0.10	0.84±0.10
Bak1	1.00±0.003	1.21±0.15	1.22±0.06	0.47±0.02
Bax	1.00±0.003	1.48±0.14	1.16±0.09	0.73±0.12 *P*=0.0191 vs. Ctrl+UVA

	24 hours after irradiation with 5 J/cm^2			
Vitamin D-Receptor (*VDR*)	1.01±0.008	0.90±0.11	0.90±0.05	0.61±0.15
P2X7-Receptor (*P2RX7*)	1.02±0.01	0.75±0.06	0.93±0.02	0.38±0.10
Bcl-2	1.01±0.005	0.67±0.03	0.92±0.12	0.70±0.08
Bak1	1.00±0.001	1.16±0.06	0.98±0.19	1.13±0.4
Bax	1.02±0.01	1.38±0.07	1.16±0.32	1.69±0.47

Data are shown as mean±SEM. For Vitamin D-Receptor (*VDR*), P2X7-Receptor (*P2RX7*)**,** *Bcl-2*, *Bak1*, and *Bax* *n*=3 biopsies per group. Data are shown as mean±SEM. For comparison of groups P-value was determined using Kruskal-Wallis with Dunn´s multiple comparisons test. Numerical source data are provided within the Supplementary Data 1 file.

Uncropped Western Blots:

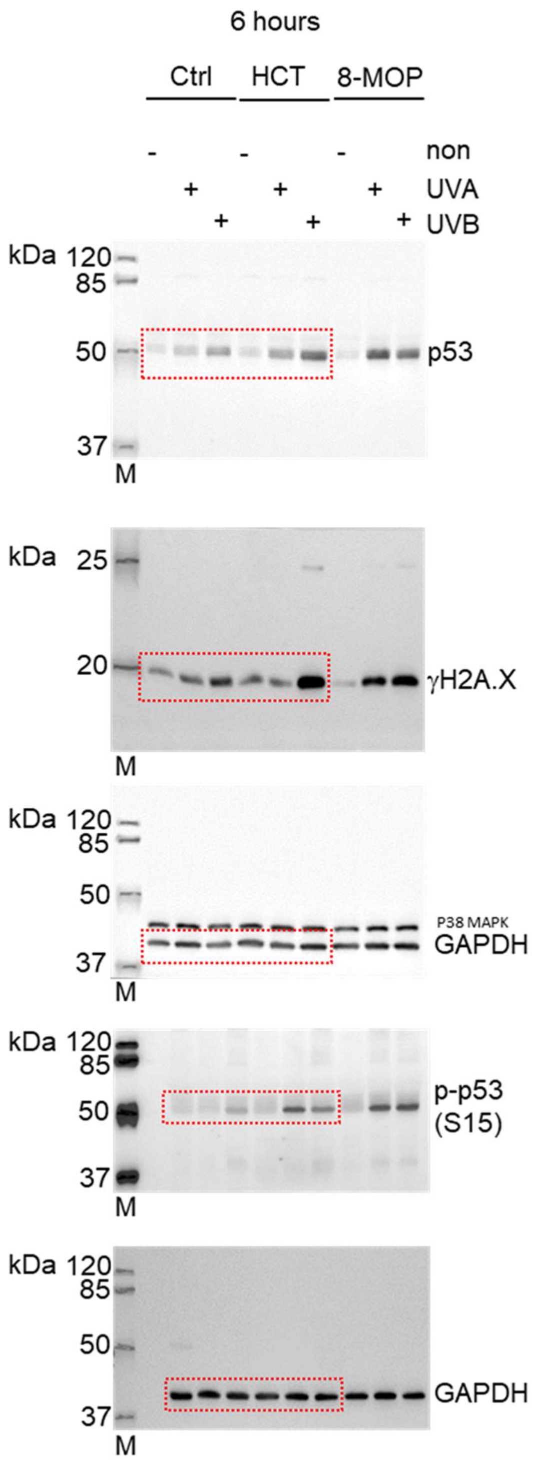

Supplementary Figure 13:
Unprocessed Western Blot for Figure 2A

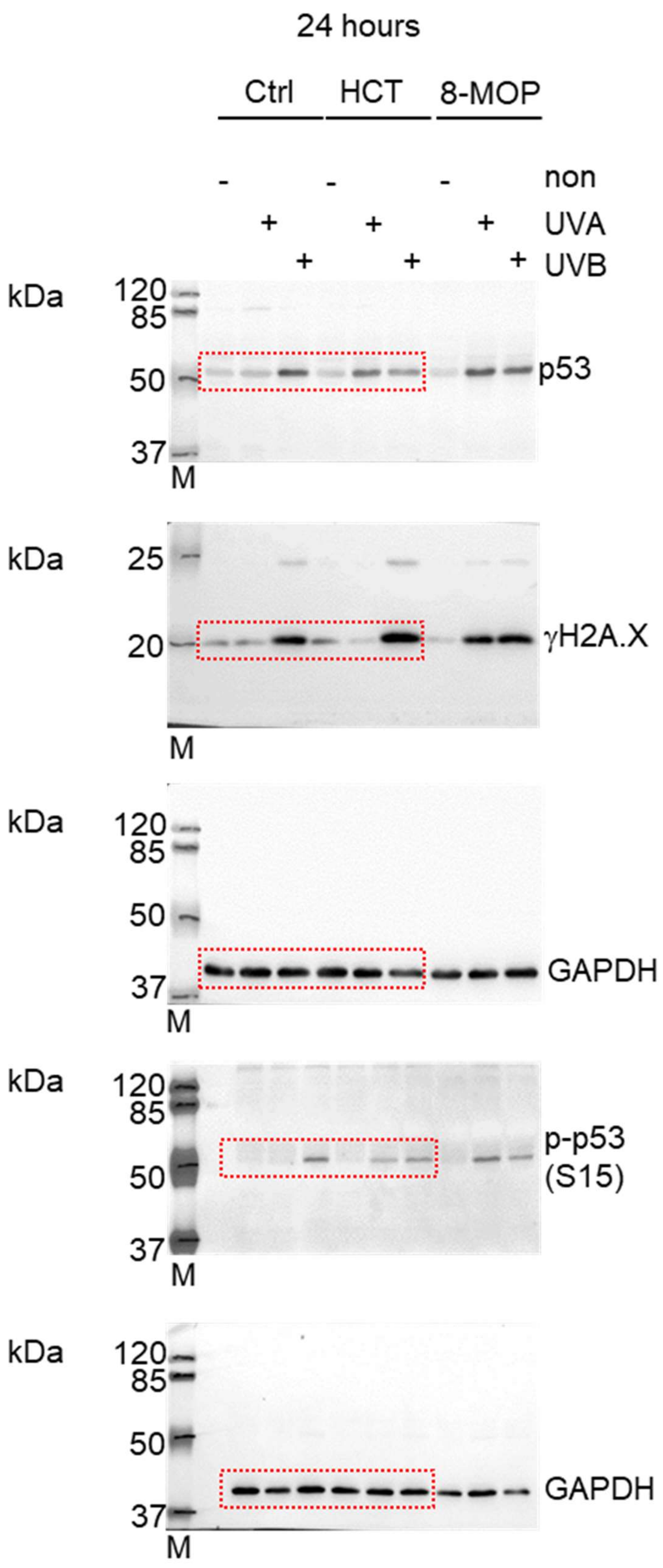

Supplementary Figure 14:
Unprocessed Western Blot for Figure 2F

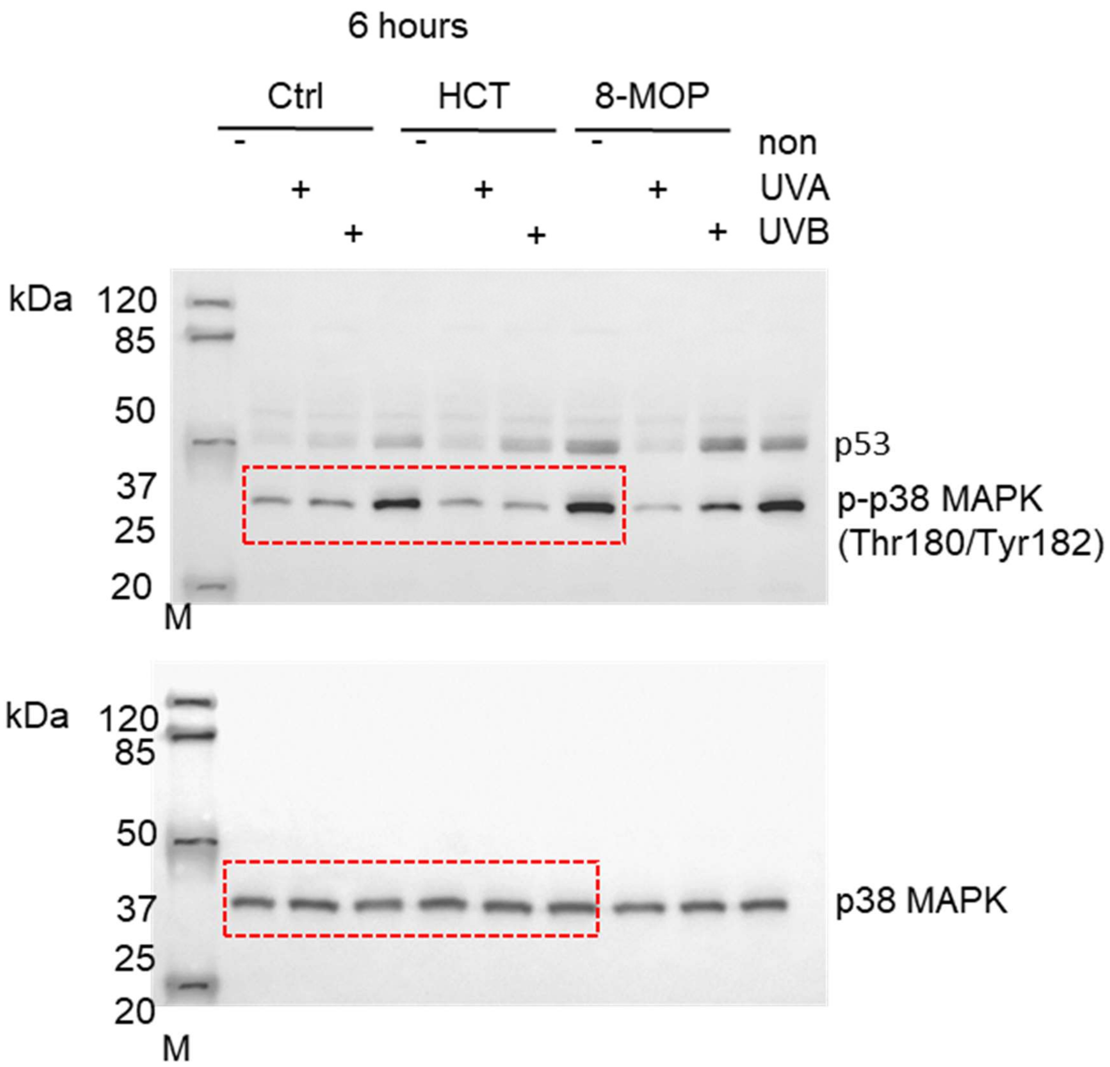

<u>Supplementary Figure 15</u>:
Unprocessed Western Blot for Figure 4A

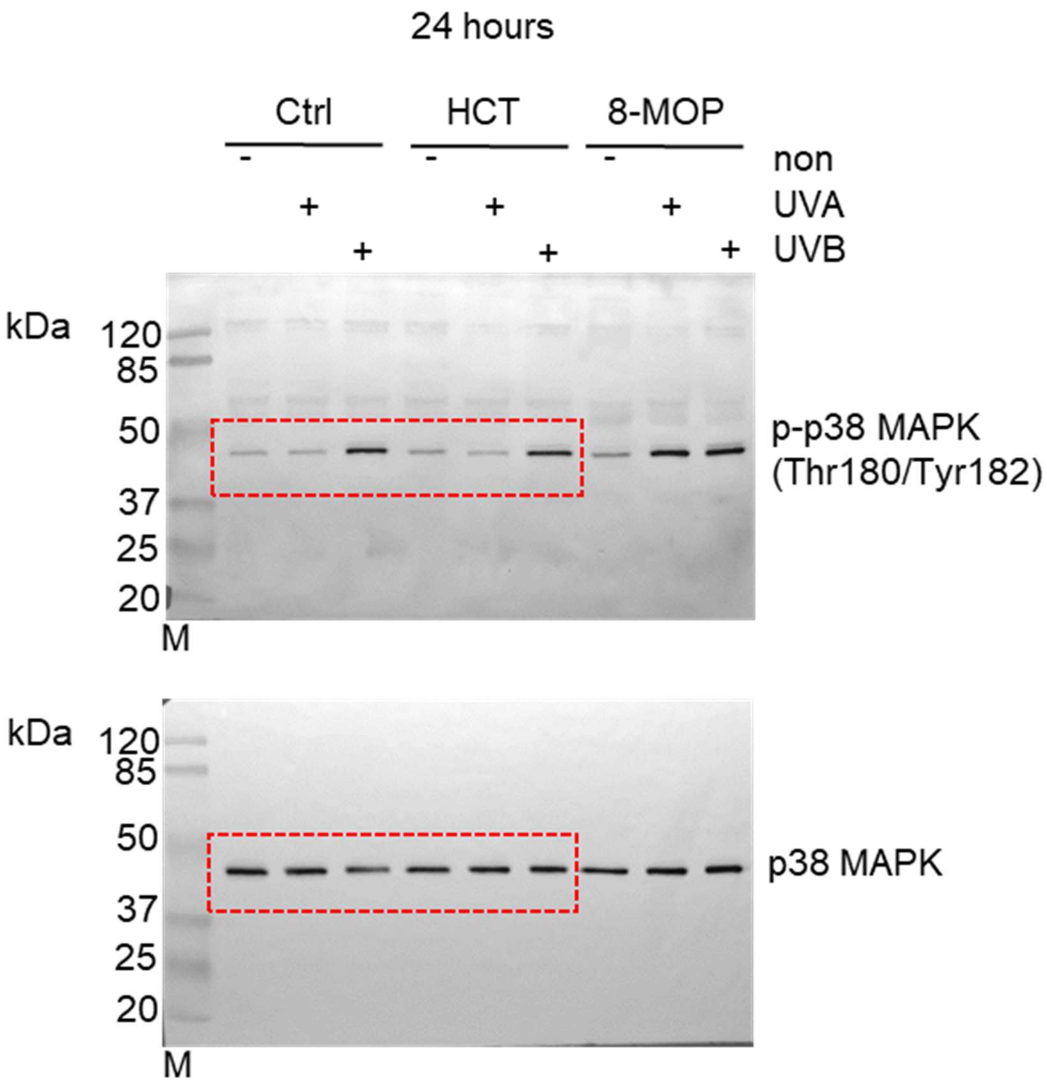

Supplementary Figure 16:
Unprocessed Western Blot for Figure 4F

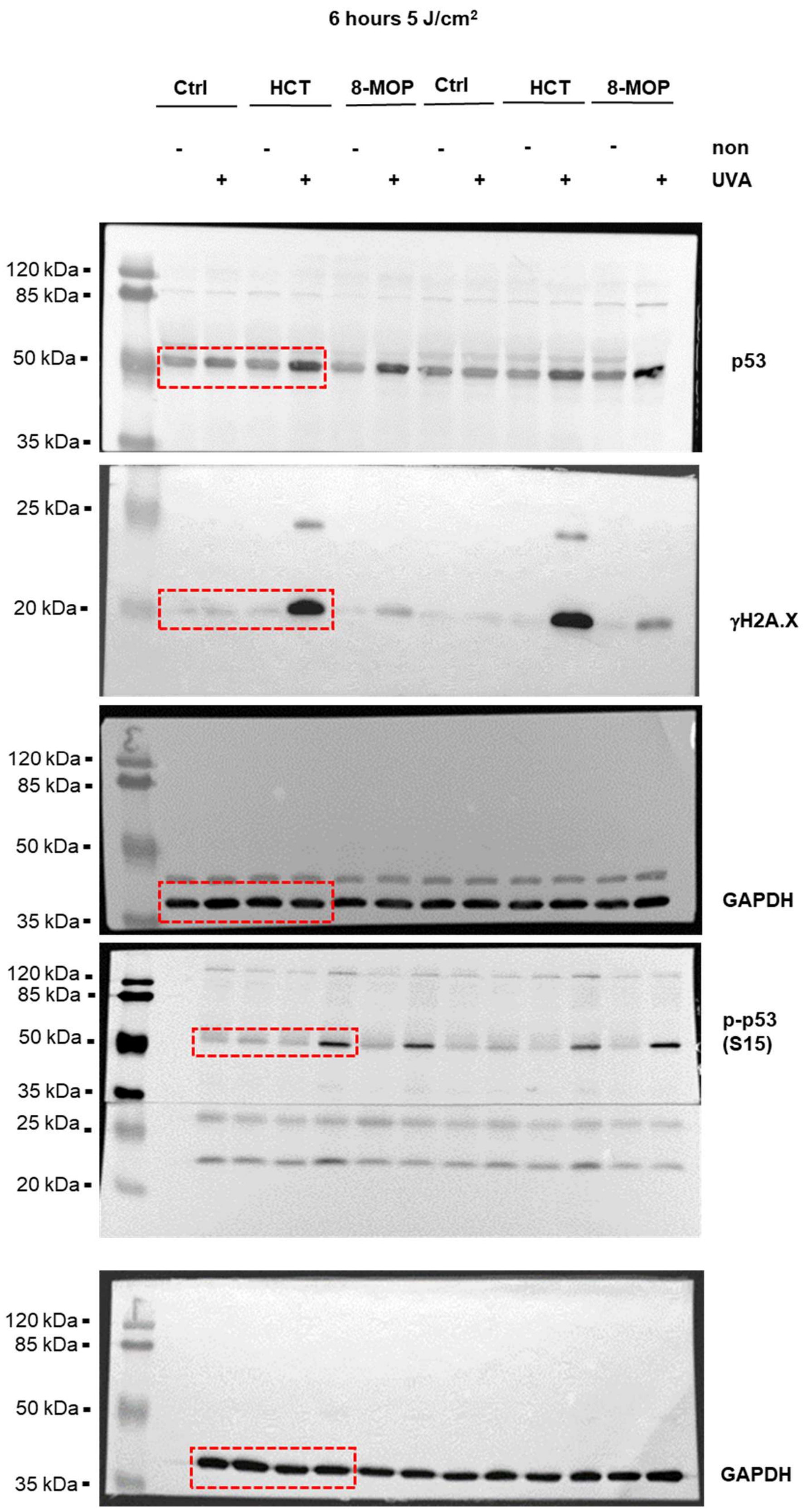

Supplementary Figure 17:
Unprocessed Western Blot for Figure 5A

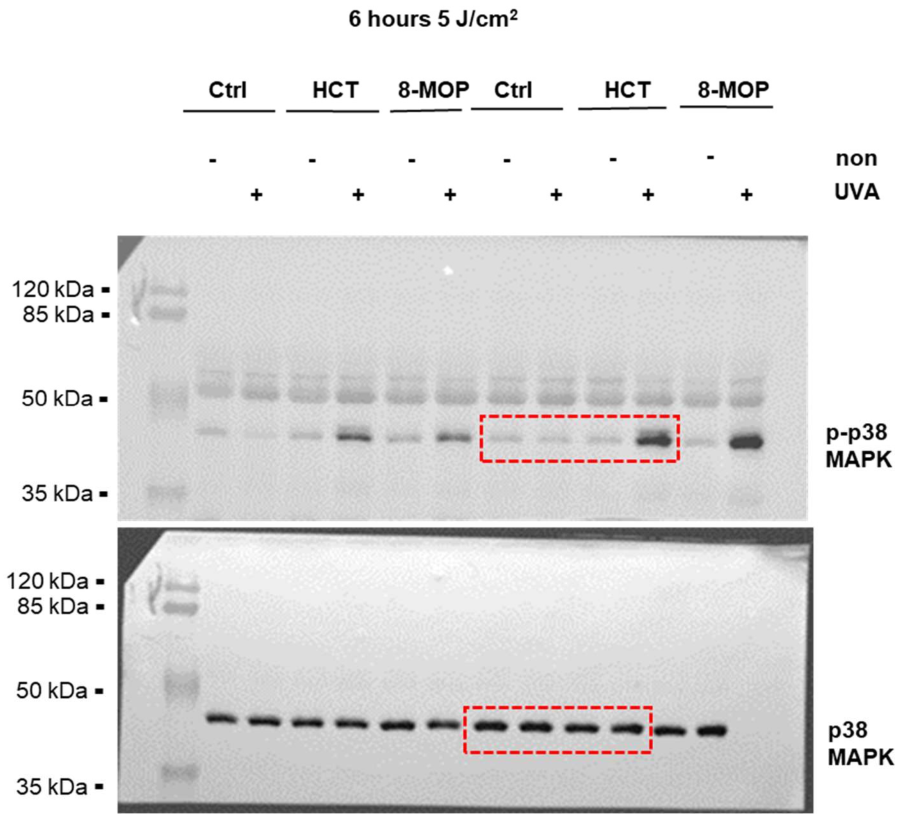

Supplementary Figure 18:
Unprocessed Western Blot for Figure 5G

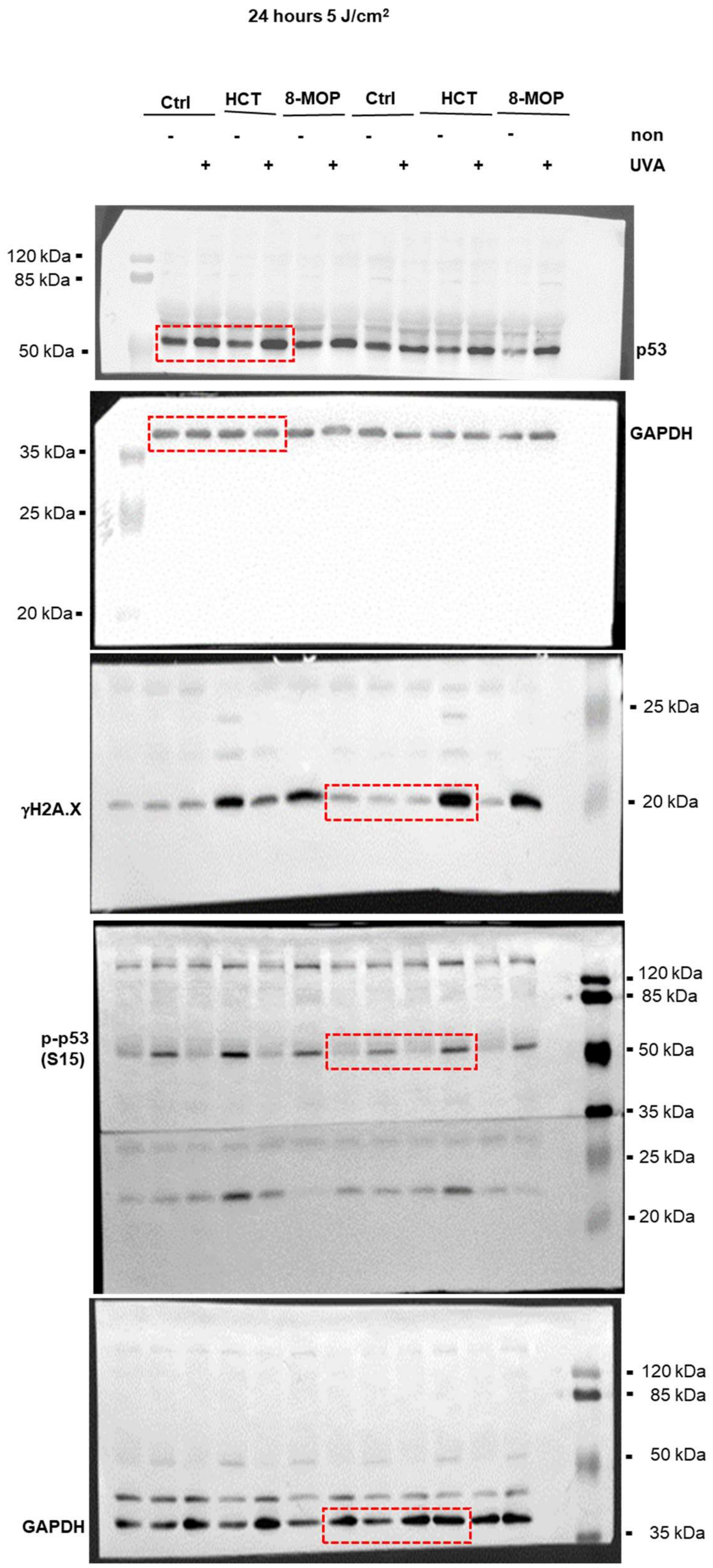

Supplementary Figure 19:
Unprocessed Western Blot for Figure 6A

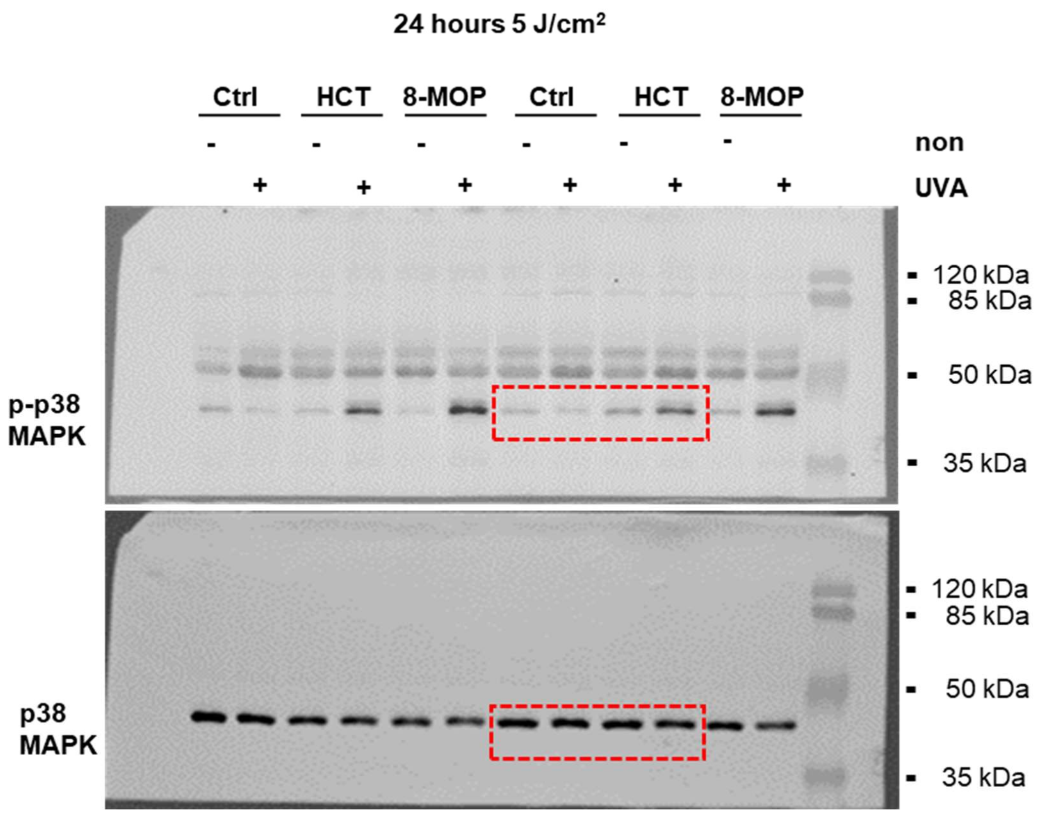

Supplementary Figure 20:
Unprocessed Western Blot for Figure 6G

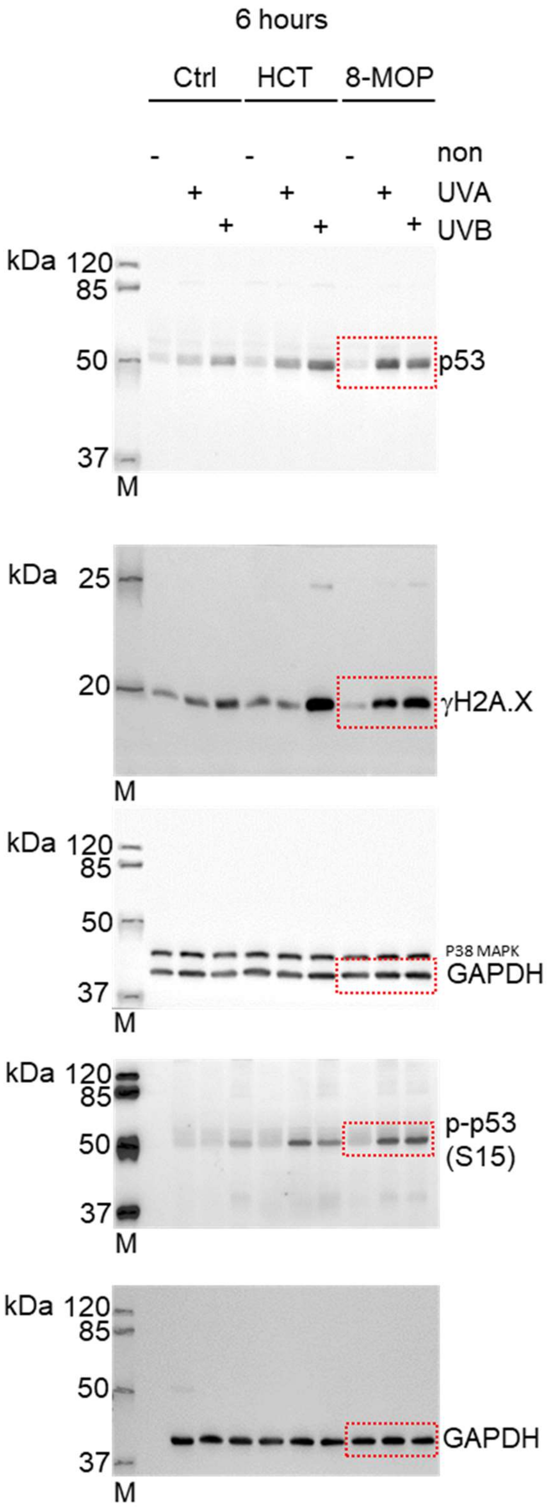

Supplementary Figure 21:
Unprocessed Western Blot for Supplementary Figure 1A

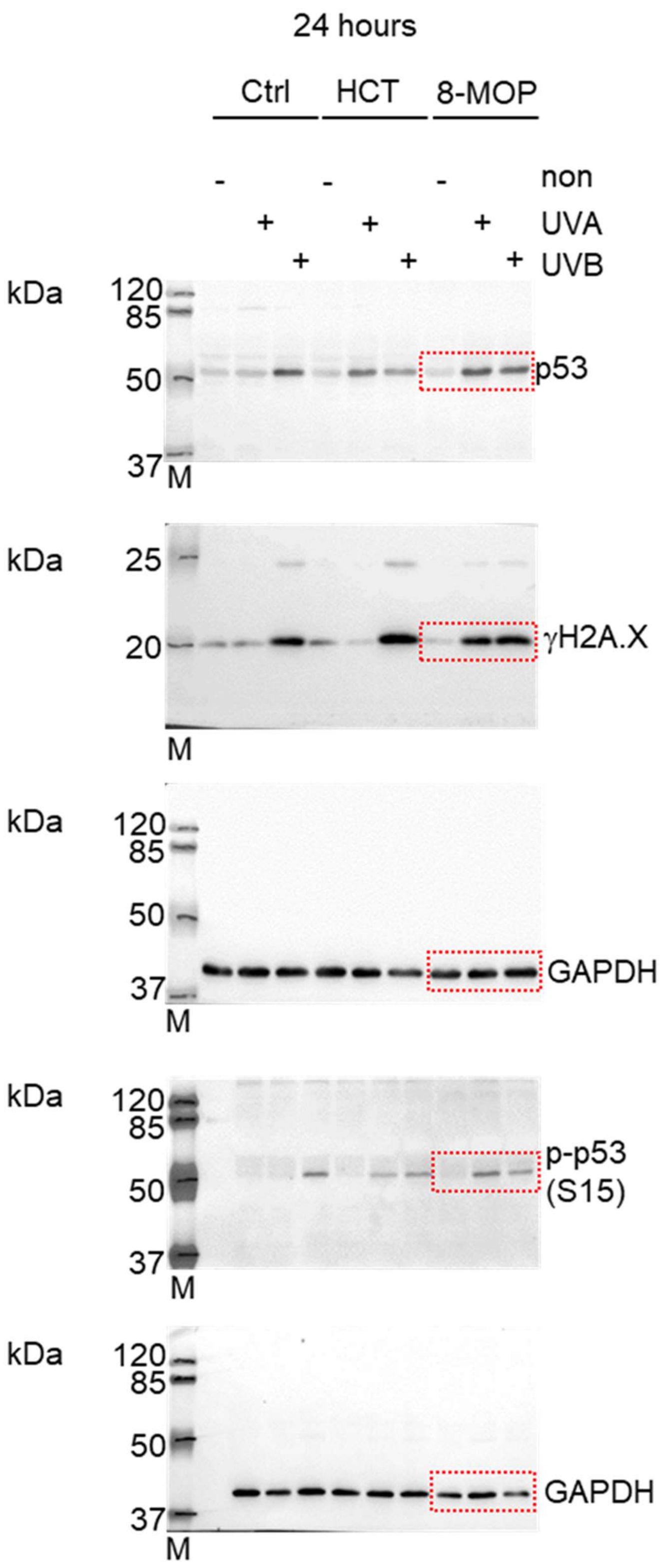

Supplementary Figure 22:
Unprocessed Western Blot for Supplementary Figure 1F

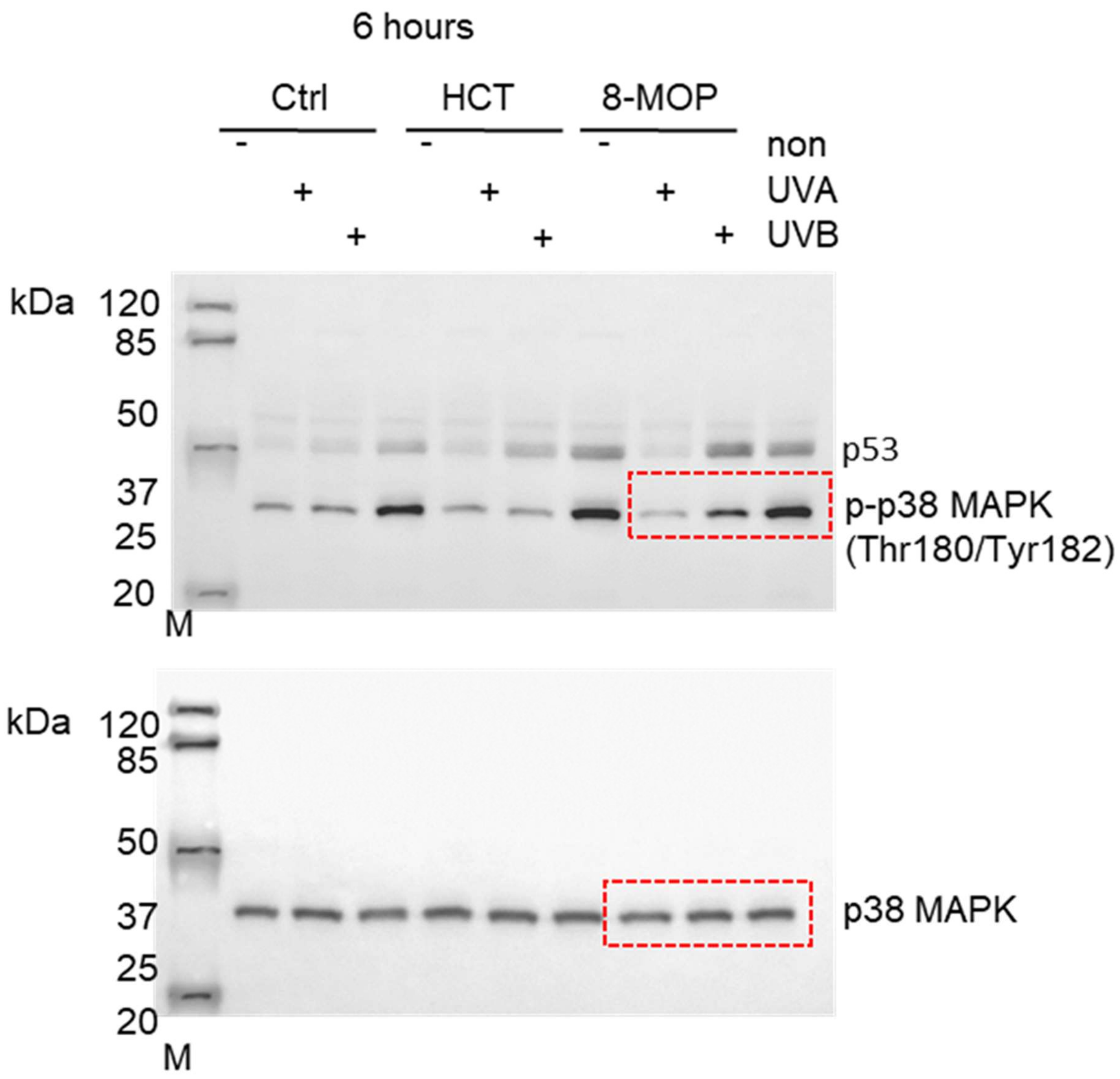

Supplementary Figure 23:
Unprocessed Western Blot for Supplementary Figure 3A

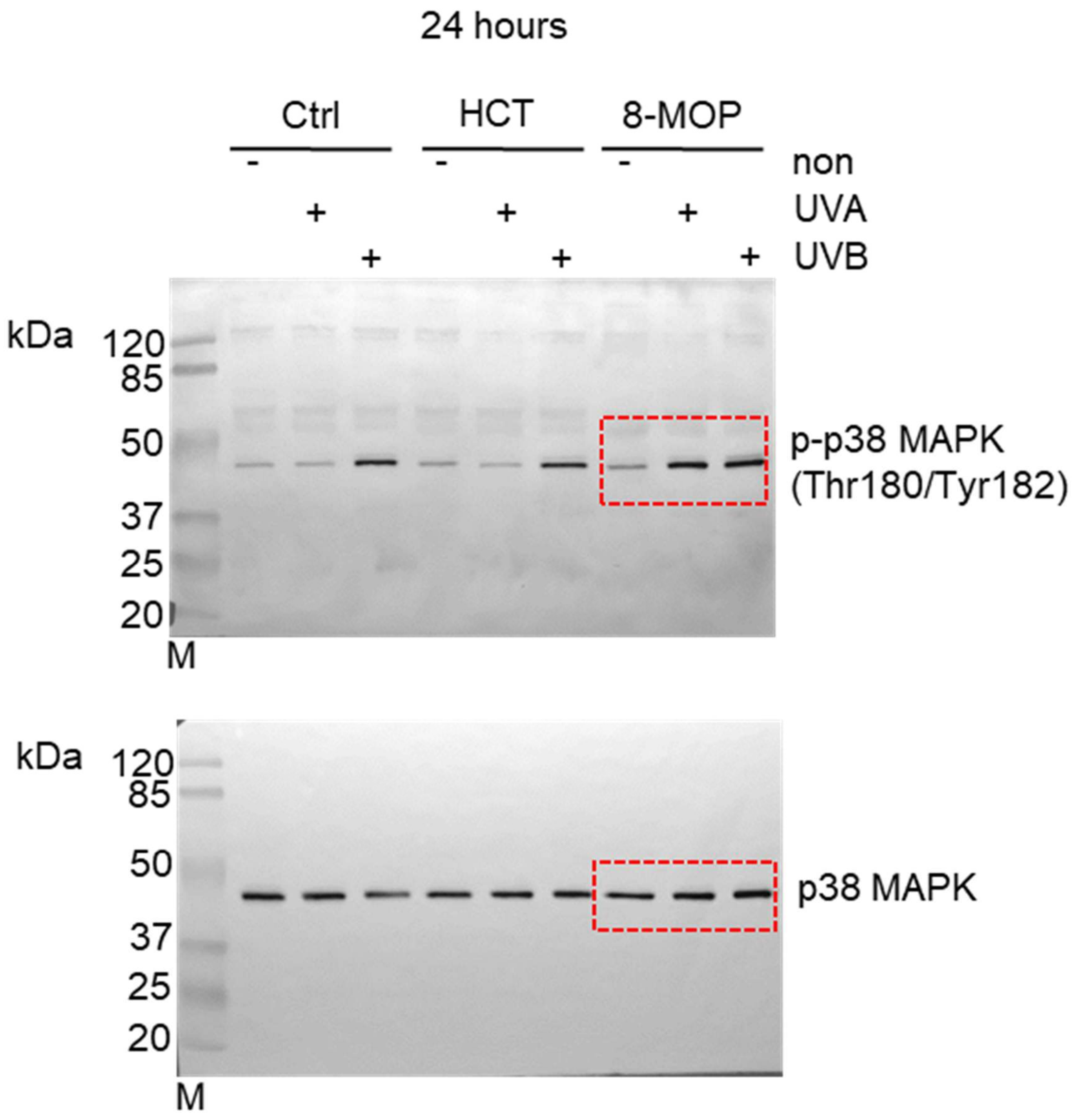

Supplementary Figure 24:
Unprocessed Western Blot for Supplementary Figure 3E

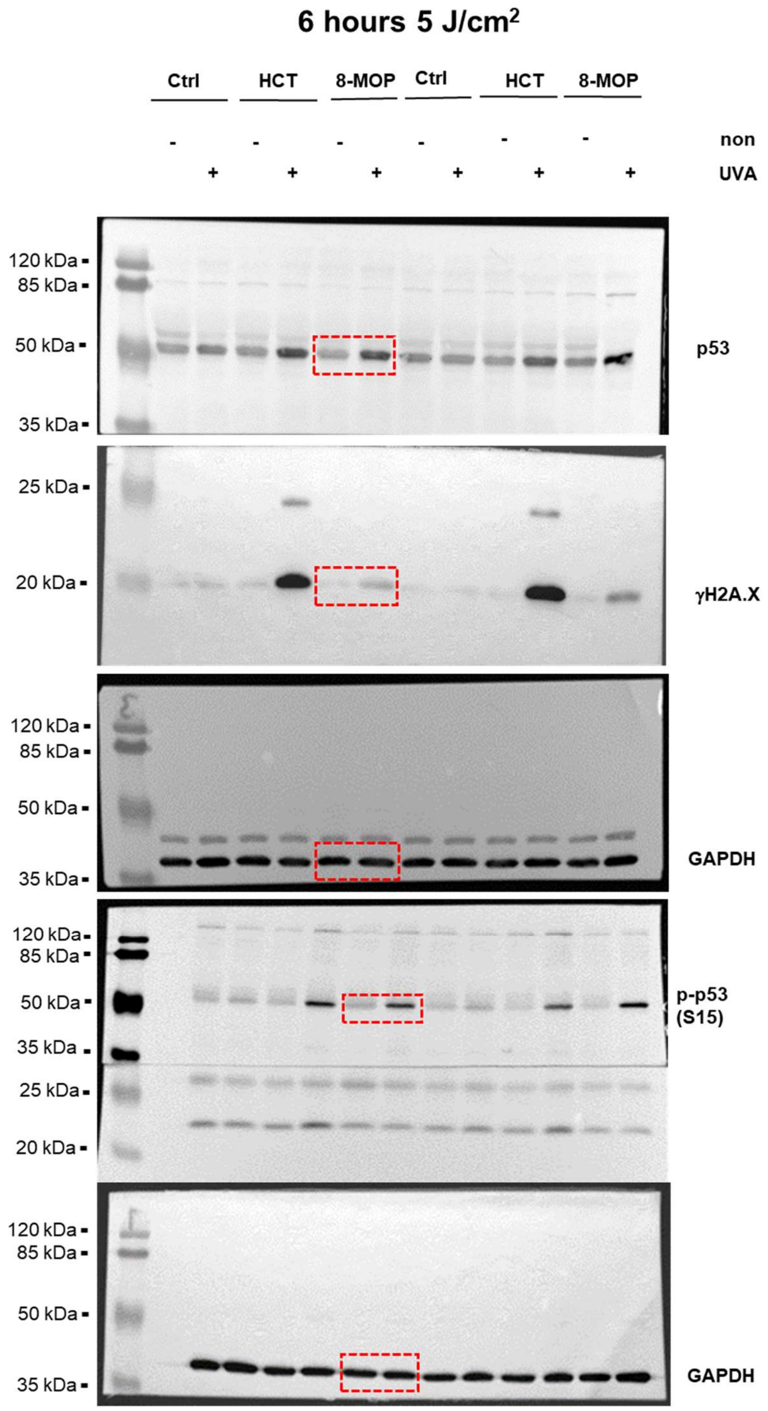

Supplementary Figure 25:
Unprocessed Western Blot for Supplementary Figure 4A

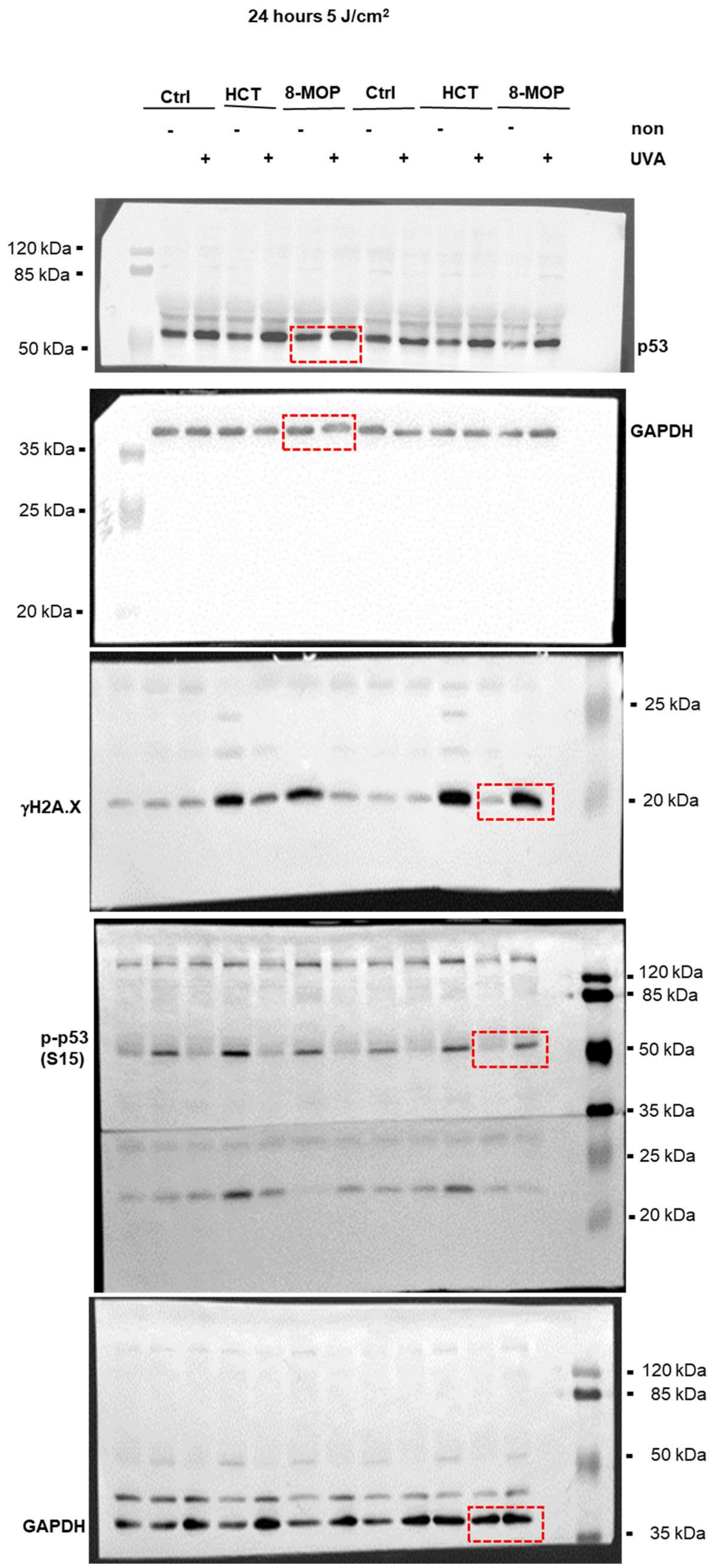

Supplementary Figure 26:
Unprocessed Western Blot for Supplementary Figure 4F

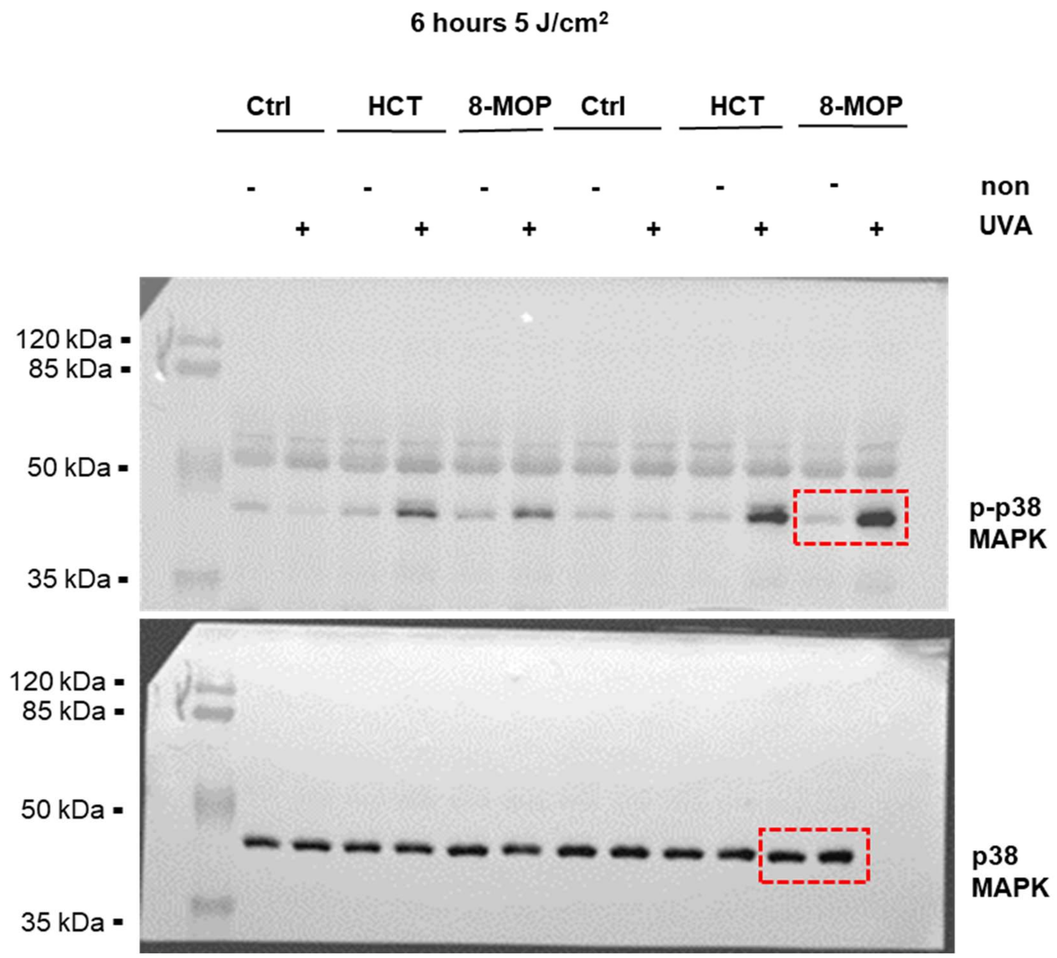

Supplementary Figure 27:
Unprocessed Western Blot for Supplementary Figure 6A

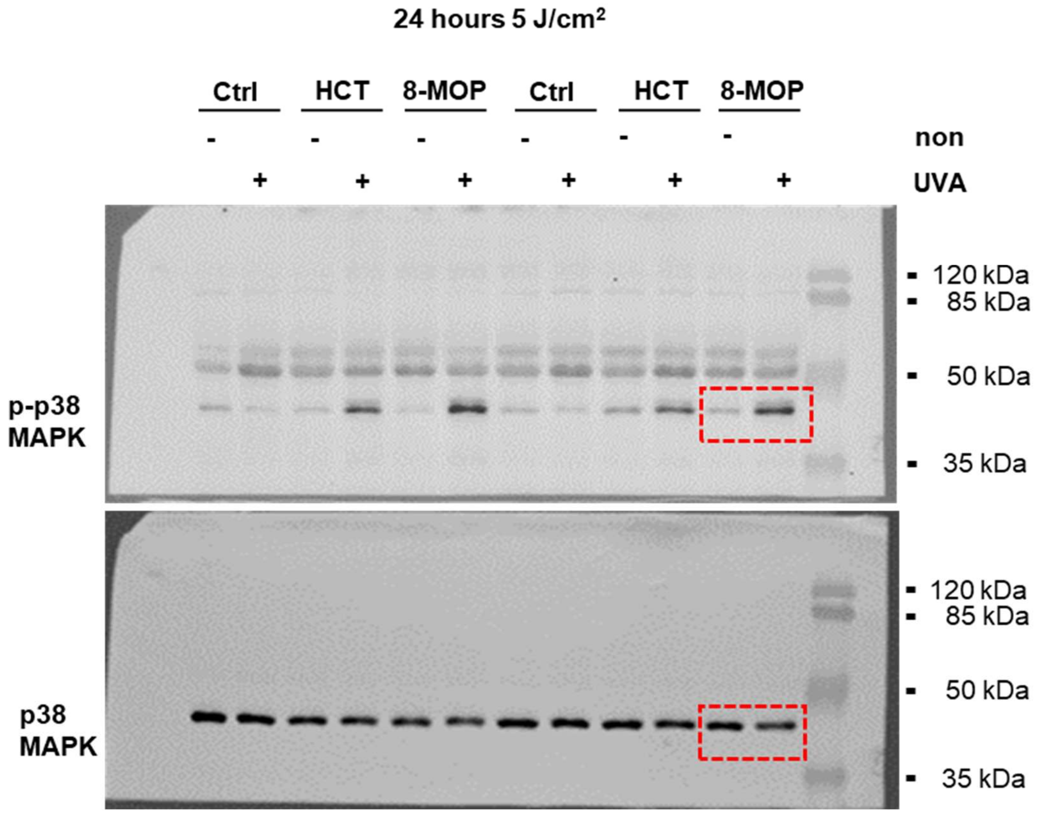

Supplementary Figure 28:
Unprocessed Western Blot for Supplementary Figure 6E
