# Supplementary material for: Assessing phototoxic drug properties of hydrochlorothiazide using human skin biopsies
Source: Commun Biol. 2025 May 6;8:705. doi: 10.1038/s42003-025-08064-1 (PMC12056033; doi:10.1038/s42003-025-08064-1)
Supplement: Supplementary file 3 — Description of Additional Supplementary Files [file 42003_2025_8064_MOESM3_ESM.docx]

Description of Additional Supplementary Files

**File name:** Supplementary Data 1

**Description:** The numerical source data behind the graphs and tables in the paper as well as the Supplemental figures and tables.
