# Supplementary material for: Assessing phototoxic drug properties of hydrochlorothiazide using human skin biopsies
Source: Commun Biol. 2025 May 6;8:705. doi: 10.1038/s42003-025-08064-1 (PMC12056033; doi:10.1038/s42003-025-08064-1)
Supplement: Supplementary file 4 — Reporting Summary [file 42003_2025_8064_MOESM4_ESM.pdf]

Corresponding author(s): Mathias Hohl

Last updated by author(s): 28-03-25

Reporting Summary

Nature Portfolio wishes to improve the reproducibility of the work that we publish. This form provides structure for consistency and transparency in reporting. For further information on Nature Portfolio policies, see our Editorial Policies and the Editorial Policy Checklist.

Statistics

For all statistical analyses, confirm that the following items are present in the figure legend, table legend, main text, or Methods section.

n/a	Confirmed	
☐	☒	The exact sample size (*n*) for each experimental group/condition, given as a discrete number and unit of measurement
☐	☒	A statement on whether measurements were taken from distinct samples or whether the same sample was measured repeatedly
☐	☒	The statistical test(s) used AND whether they are one- or two-sided<br>*Only common tests should be described solely by name; describe more complex techniques in the Methods section.*
☒	☐	A description of all covariates tested
☐	☒	A description of any assumptions or corrections, such as tests of normality and adjustment for multiple comparisons
☐	☒	A full description of the statistical parameters including central tendency (e.g. means) or other basic estimates (e.g. regression coefficient) AND variation (e.g. standard deviation) or associated estimates of uncertainty (e.g. confidence intervals)
☒	☐	For null hypothesis testing, the test statistic (e.g. *F*, *t*, *r*) with confidence intervals, effect sizes, degrees of freedom and *P* value noted<br>*Give P values as exact values whenever suitable.*
☒	☐	For Bayesian analysis, information on the choice of priors and Markov chain Monte Carlo settings
☒	☐	For hierarchical and complex designs, identification of the appropriate level for tests and full reporting of outcomes
☒	☐	Estimates of effect sizes (e.g. Cohen's *d*, Pearson's *r*), indicating how they were calculated

Our web collection on statistics for biologists contains articles on many of the points above.

Software and code

Policy information about availability of computer code

Data collection	Aperio ImageScope x64 whole slide scanner (Leica biosystems, Aperio ImageScope Wetzlar, Germany) was used for image acquisition<br>Vilber Loumat Fusion Solo S documentation system (Evolution-Capt Edge) (AvantorTM, VWRTM, Germany) was used for Western Blot images aquisition<br>TaqMan PCR was conducted in a StepOne plus thermocycler (Applied Biosystems, Waltham, USA)<br>Dionex Ultimate UHPLC System (Thermo Fisher, TF, Dreieich, Germany) was used to determine bioavailability of HCT in biopsies
Data analysis	Leica biosystems, Aperio ImageScope (Wetzlar, Germany) was used for image analysis<br>Vilber Loumat Fusion Solo S documentation system (Evolution-Capt Edge) (AvantorTM, VWRTM, Germany) was used for Western Blot analysis<br>Statistical analysis was performed using GraphPad Prism 10.1.2.

For manuscripts utilizing custom algorithms or software that are central to the research but not yet described in published literature, software must be made available to editors and reviewers. We strongly encourage code deposition in a community repository (e.g. GitHub). See the Nature Portfolio guidelines for submitting code & software for further information.

Data

Policy information about availability of data

All manuscripts must include a data availability statement. This statement should provide the following information, where applicable:

- Accession codes, unique identifiers, or web links for publicly available datasets
- A description of any restrictions on data availability
- For clinical datasets or third party data, please ensure that the statement adheres to our policy

All data supporting the findings of this study are available within the paper, its Supplementary Information, and within the source data file

Research involving human participants, their data, or biological material

Policy information about studies with human participants or human data. See also policy information about sex, gender (identity/presentation), and sexual orientation and race, ethnicity and racism.

Reporting on sex and gender	Skin biopsies of six female and three male were collected
Reporting on race, ethnicity, or other socially relevant groupings	All body donors were caucasians
Population characteristics	n.a
Recruitment	n.a
Ethics oversight	The ethical committee of the Medical Association of Saarland approved the study (number 162/20)

Note that full information on the approval of the study protocol must also be provided in the manuscript.

Field-specific reporting

Please select the one below that is the best fit for your research. If you are not sure, read the appropriate sections before making your selection.

☒ Life sciences ☐ Behavioural & social sciences ☐ Ecological, evolutionary & environmental sciences

For a reference copy of the document with all sections, see nature.com/documents/nr-reporting-summary-flat.pdf

Life sciences study design

All studies must disclose on these points even when the disclosure is negative.

Sample size	Exact sample size is given in the manuscript. No statistical method was used to predetermine the sample size.
Data exclusions	no data were excluded
Replication	Replication was susessful. Additionally, a well described positive control (8-MOP) was used and run in paralell
Randomization	The experiments were not randomized.
Blinding	The investigators were not blinded when performing and analyzing histological immunostaining, western blot and quantitative PCR.

Reporting for specific materials, systems and methods

We require information from authors about some types of materials, experimental systems and methods used in many studies. Here, indicate whether each material, system or method listed is relevant to your study. If you are not sure if a list item applies to your research, read the appropriate section before selecting a response.

Materials & experimental systems

n/a	Involved in the study
☐	☒ Antibodies
☒	☐ Eukaryotic cell lines
☒	☐ Palaeontology and archaeology
☒	☐ Animals and other organisms
☒	☐ Clinical data
☒	☐ Dual use research of concern
☒	☐ Plants

Methods

n/a	Involved in the study
☒	☐ ChIP-seq
☒	☐ Flow cytometry
☒	☐ MRI-based neuroimaging

Antibodies

Antibodies used

For Wesstern blotting:
Anti-yH2A.X (phospho S139), rabbit monoclonal Ab, Abcam, #ab81299), diluted (1:1000)
Anti-p53 rabbit Ab (#9282S; Cell Signaling Technologies), diluted (1:1000)
Anti-phospho-p53 (S15) rabbit Ab (#9284S; Cell Signaling Technologies) diluted (1:1000)
Anti-p38 MAPK rabbit Ab (#9212S; Cell Signaling Technologies) diluted (1:1000)
Anti-phospho-p38 MAPK (T180/Y182) (12F8) rabbit Ab (#4631S; Cell Signaling Technologies) diluted (1:1000)
Anti-glyceraldehyde-3-phosphate dehydrogenase (GAPDH; MAB374, Millipore, Darmstadt, Germany) diluted (1:10000)
Anti-Catalase rabbit mAb (#14097S, Cell Signaling Technologies, diluted (1:1000)
Anti-superoxide dismutase 1 (SOD-1) rabbit polyclonal IgG (sc-11407, Santa Cruz Biotechnology, diluted 1:5000)
Anti-superoxide dismutase 2 (SOD-2) rabbit polyclonal IgG (sc-30080, Santa Cruz Biotechnology, diluted 1:5000)
For Immunohistological stainings:
Anti-p53 (DO-7) mouse mAb, (#48818S; Cell Signaling Technologies), diluted (1:100)
Anti-yH2A.X (phospho S139) [EP854(2)Y], rabbit monoclonal Ab, Abcam, #ab81299), diluted (1:100)

Validation

All Antibodies were purchased from Abcam, Cell Signaling Technologies or Sigma-Aldrich:
Informations on validations can be found on the respective website
https://www.abcam.com/en-us/products/primary-antibodies/gamma-h2ax-phospho-s139-antibody-ep8542y-ab81299
https://www.cellsignal.com/products/primary-antibodies/p53-antibody/9282
https://www.cellsignal.com/products/primary-antibodies/phospho-p53-ser15-antibody/9284
https://www.cellsignal.com/products/primary-antibodies/p38-mapk-antibody/9212
https://www.cellsignal.com/products/primary-antibodies/phospho-p38-mapk-thr180-tyr182-12f8-rabbit-mab/4631
https://www.sigmaaldrich.com/DE/de/product/mm/mab374?
utm_source=google&utm_medium=cpc&utm_id=21480163361&utm_campaign=%7Bcampaignname%
7D&utm_content=167683195609&utm_term=gapdh+millipore+mab374&gclid=EAIaIQobChMImNvE-c-
QiQMVRUdHAR0D9ibHEAAYAiAAEgIrJPD_BwE
https://www.cellsignal.com/products/primary-antibodies/catalase-d5n7v-rabbit-mab/14097
https://www.scbt.com/p/sod-1-antibody-fl-154
https://www.scbt.com/p/sod-2-antibody-fl-222
https://www.cellsignal.com/products/primary-antibodies/p53-do-7-mouse-mab/48818?
srsltid=AfmBOopOhV8tjKhrA0WKIk47FNujAKeuXusMMyLc3CG8WY242cJqj8le

Plants

Seed stocks

n.a

Novel plant genotypes

n.a

Authentication

n.a
